# Tailored and Interactive Mobile Telehealth Contraceptive Counseling Compared With In-Person Care: Systematic Review and Meta-Analysis of Randomized Controlled Trials

**DOI:** 10.2196/88887

**Published:** 2026-07-16

**Authors:** Maja Weinryb, Wilhelmus JA Grooten, Andrea Koris, Margit Endler

**Affiliations:** 1 Department of Women's and Children's Health Karolinska Institutet Stockholm, Stockholm Sweden; 2 Division of Social and Behavioural Sciences School of Public Health University of Cape Town Cape Town, Western Cape South Africa; 3 Department of Neurobiology, Care Sciences and Society Division of Physiotherapy Karolinska Institutet Stockholm Sweden; 4 School of Health and Welfare Dalarna University Dalarna Sweden; 5 Global Women's Institute George Washington University Washington, DC United States

**Keywords:** contraceptive counseling, systematic review, telemedicine, randomized controlled trials, meta-analysis, long-acting reversible contraception, mobile health

## Abstract

**Background:**

Use of effective contraceptive methods (ECMs) reduces maternal mortality. Person-centered counseling increases uptake, but barriers to high-quality counseling persist. Telehealth may improve access to comprehensive contraceptive care, but its effectiveness remains unclear.

**Objective:**

This study assesses the effectiveness and acceptability of tailored, interactive telehealth contraceptive counseling (TECC) compared with in-person counseling.

**Methods:**

We conducted a systematic review and meta-analysis of English-language randomized controlled trials (RCTs) comparing TECC with in-person counseling for women and girls of any age and setting. We searched MEDLINE, Embase, Web of Science, and the Cochrane Library from inception through October 15, 2025. Outcomes were use of ECM less than 6 months from intervention (primary outcome); use of ECM at 6-12 months; use of long-acting reversible contraception (LARC); choice of ECM; choice of LARC; satisfaction with counseling; and method switching. Two researchers assessed risk of bias (RoB) using Cochrane RoB 2, certainty of evidence using GRADE (Grading of Recommendations Assessment, Development, and Evaluation), and performed meta-analysis using a random-effects model. The protocol was registered with PROSPERO (International Prospective Register of Systematic Reviews) a priori (CRD42023404402). No specific funding was received.

**Results:**

Eight RCTs (RoB: low, n=4; moderate, n=2; and high, n=2) and 1 cluster RCT (moderate-to-high RoB) were included in the review, with 5353 participants across all included studies. Eight studies evaluated TECC as an adjunct to in-person care (hybrid model) and 1 as a standalone model, of which 7 and 1, respectively, contributed outcome data for the meta-analyses. Certainty of evidence was low to very low. The pooled effect of 4 studies of TECC on ECM use less than 6 months showed no clear evidence of an effect (relative risk [RR] 1.10; 95% CI 0.95-1.29). The pooled effect of 4 studies of TECC on choice of ECM (RR 1.07; 95% CI 0.96-1.18) likewise showed no clear effect. The pooled effect of 4 studies of TECC on ECM use at 6-12 months showed a small but statistically significant (*P*=.04) effect in favor of TECC, narrowly excluding the null (RR 1.07; 95% CI 1.002-1.130). Because of high statistical and clinical heterogeneity (I2=87%-96%), results for LARC choice (2 studies) and LARC use at 0-6 months (2 studies) and 6-12 months (2 studies) were narratively described. The evidence for these outcomes was very uncertain. Narrative analysis of satisfaction across 2 studies showed no difference in effect. There were no data on method switching to support the analysis.

**Conclusions:**

Current evidence suggests, with low certainty, that adjunct TECC, when delivered alongside in-person care, shows little to no effect on contraceptive use compared with in-person care. For use at 12 months, method choice, LARC use, and satisfaction compared with in-person care, the evidence is very uncertain. Future research should prioritize adequately powered evaluations of standalone models and assess how tailoring, timing, and delivery influence effectiveness, including long-term use and method switching.

## Introduction

Contraception is one of the most cost-effective public health strategies for reducing maternal mortality and improving reproductive, perinatal, and child health. Many unintended pregnancies and maternal deaths globally could be averted by satisfying the unmet need for modern contraceptives [1-3]. Women and girls continue to face barriers to access due to misconceptions and concerns about side effects, social stigma, provider bias, geographic barriers, and health system constraints [4]. Barriers disproportionately affect already vulnerable populations. Person-centered contraceptive counseling (CC) aims to support informed contraceptive decision-making by addressing misconceptions and side effects and tailoring counseling to the individual’s preferences, values, needs, medical eligibility, and reproductive life goals. High-quality counseling has been shown to improve the use and continuation of effective modern contraception [5].

The core components of person-centered CC, such as eliciting individual preferences and addressing misconceptions and side effect concerns, could in principle be delivered remotely through interactive telehealth formats. The WHO defines telehealth as the use of electronic information and communication technologies for the “delivery of health care services, where patients and providers are separated by distance” [6]. Telehealth can offer a convenient, low-cost, and private alternative to in-person care. It can also expand access to person-centered care in settings where geographic or system constraints limit access through messaging, web-based tools, mobile apps, or virtual consultations [7].

Telehealth contraception services have been used for more than a decade, with commercial services expanding in response to technological advances and the COVID-19 pandemic. These digital services range from prescription and delivery services, 1-way messaging, and decision aids to more comprehensive counseling delivered by telephone or video. Several US-based observational studies report high acceptability, but evidence of effectiveness remains limited and heterogeneous [8-12]. Previous reviews have grouped heterogeneous digital contraceptive services, including 1-way messaging and nontailored models. However, none have specifically examined tailored and interactive telehealth contraceptive counseling (TECC) [11-15].

Our definition of TECC entails bidirectional communication and individualized content aligned with users’ preferences, reproductive goals, and medical eligibility. Although TECC can conceptually function as a standalone counseling modality, most existing telehealth contraception trials have evaluated hybrid models delivered as an adjunct to in-person care, and the extent to which TECC can substitute for in-person counseling remains unknown. We aimed to systematically assess the effectiveness and acceptability of standalone and hybrid TECC compared with any form of standard in-person contraceptive care, including verbal or written information delivered individually or in a group setting.

## Methods

### Search Strategy

We followed the PRISMA (Preferred Reporting Items for Systematic Reviews and Meta-Analyses) guidelines (see Multimedia Appendix 1) [16]. The protocol was registered a priori with PROSPERO (International Prospective Register of Systematic Reviews; registration number CRD42023404402) [17]. We searched MEDLINE, Web of Science, Embase, the Cochrane Library from inception to October 15, 2025, using MeSH (Medical Subject Headings) and free-text terms, with a validated randomized controlled trial (RCT) filter. A snowball search was conducted to identify references cited by and citations of eligible studies identified through the database searches. The full search strategies for all databases are available in Multimedia Appendix 2.

### Eligibility Criteria

We included single and cluster RCTs comparing TECC with routine in-person CC among women and girls of any age or setting. Only full, published, English-language reports were eligible; abstracts and study protocols were excluded. Intervention eligibility required meeting 3 core domains: telehealth delivery modality, interactivity, and tailoring. Telehealth delivery modalities included phone or video calls, voice or SMS text messaging, mobile apps, and websites. Interactivity implied bidirectional communication to some degree, excluding, for example, unidirectional information delivery or reminders. Tailoring meant that interventions were individualized to users’ input, generating adapted output based on at least one of the following domains: contraceptive preferences, reproductive goals, medical history, or method-specific information on benefits, side effects, and effectiveness. Interventions could be delivered in real time or asynchronously, with or without human involvement. Interventions could be delivered before, during, or after a clinical visit and could complement or substitute for in-person clinical care. We categorized models in which TECC was delivered as an adjunct to in-person care as hybrid models. Hybrid models were eligible if the telehealth component independently met the tailoring and interactivity criteria. Shared decision-making tools used in provider-led consultations were excluded. Our comparator was any form of routine CC delivered in person as verbal or written information, provided individually or in groups. Studies using telehealth as a comparator were excluded. The rationale for excluding other digital interventions as comparators was to assess the effect of the telehealth component on CC. An overview of the concepts in our definition of TECC is provided in Multimedia Appendix 3.

### Outcomes

Our primary outcome was the use of any effective contraceptive method (ECM) up to 6 months after the intervention. We distinguished use from continuation or adherence to a specific method. ECM was defined as long-acting reversible contraception (LARC), including the progestin implant and hormonal or copper intrauterine devices; combined oral contraceptives; progestin-only pills; progestin injections; the contraceptive patch; the contraceptive ring; and sterilization—methods with typical-use failure rates of less than 10% per year [18]. Additional outcomes were (1) ECM use at 6-12 months; (2) LARC use at less than 6 months; (3) LARC use at 6-12 months; (4) choice of ECM at the time of counseling; (5) choice of LARC at the time of counseling; (6) satisfaction with counseling at less than 6 months; and (7) method switching.

“Use” was assessed based on self-reported or recorded use in follow-up surveys or clinical records. Where multiple time points were reported, we prioritized the time point that enabled comparison across the greatest number of studies or, secondarily, the latest time point within the specified range. “Choice” was defined as documented intention to use a specific method immediately after counseling. We defined “switching” as a documented change from no method or a less effective method to a more effective ECM. “Satisfaction” included any reported measure of satisfaction with counseling up to 6 months after the intervention.

### Study Selection

After deduplication of the final search results, we imported records into Rayyan for screening. Three reviewers (MW, AK, and ME) independently screened titles and abstracts. We unblinded the selection process after the initial screening and resolved discrepancies through discussion. We retrieved and assessed full-text articles against the inclusion criteria, and disagreements were resolved through discussion until consensus was reached. Records excluded at the full-text review stage and the reasons for exclusion are provided in Multimedia Appendix 4.

### Data Collection and Quality Assessment

Data on publication year, study design, setting, participant characteristics, intervention, comparator, and raw data for each outcome were extracted by one reviewer (MW) from full-text articles, supported by GPT-4o (OpenAI, May 2024) as a preliminary extraction tool for nonoutcome study characteristics (population, setting, intervention, and comparator descriptions). All AI-assisted outputs were reviewed and corrected against the source articles by MW and then verified by ME. Studies in which data relevant to our outcomes were grouped in a way that did not permit the extraction of stratified data, or in which outcome data were missing, were excluded from the analysis.

Risk of bias (RoB) was assessed independently by reviewers MW and ME for each outcome using the Cochrane RoB 2 tools for RCTs and cluster RCTs, respectively [19]. Five domains were evaluated, including (1) bias arising from the randomization process, (2) bias due to deviations from intended interventions, (3) bias due to missing outcome data, (4) bias in outcome measurement, and (5) bias in the selection of the reported result. Discrepancies were resolved through discussion until consensus was reached.

Assessment of the overall certainty of evidence was performed using the GRADE (Grading of Recommendations Assessment, Development, and Evaluation) approach for each outcome. GRADE ratings were independently assessed by MW and ME; discrepancies were resolved through discussion until consensus was reached [20]. As we included only RCTs, the certainty of evidence started as high and was downgraded based on RoB, inconsistency, imprecision, indirectness, and publication bias. Statistical heterogeneity was quantified using the *I*^2^ statistic in accordance with the Cochrane Handbook for Systematic Reviews of Interventions, with 50%-90% potentially representing substantial heterogeneity and 75%-100% representing considerable heterogeneity. Our interpretation was informed by the magnitude and direction of effects, the number and size of contributing studies, and the plausibility of clinical or methodological diversity. We considered downgrading for inconsistency if *I*^2^ exceeded 50%. Imprecision was assessed using the optimal information size (OIS) criterion, in accordance with GRADE guidance for systematic reviews [21]. For each outcome eligible for meta-analysis, OIS was determined using the reference values reported by Guyatt et al [21], applying the observed pooled control event rate and the GRADE-recommended default relative risk (RR) reduction of 25%, with α=.05 and power of 80%. Certainty of evidence was downgraded by 1 level for imprecision when the total sample size contributing to the pooled estimate fell below the corresponding OIS threshold. Where the OIS threshold was met but the 95% CI of the pooled effect crossed or narrowly excluded the line of no effect, we additionally considered whether the CI excluded an appreciable effect, using the GRADE default RR reduction threshold of 25% for appreciable benefit or harm. For outcomes not eligible for meta-analysis (LARC use at 6-12 months, choice of LARC, and satisfaction with counseling), imprecision was judged by comparing the combined sample size across contributing studies with the OIS reference and by inspecting the width of individual study CIs in relation to the line of no effect. Imprecision judgments for each outcome are documented in the footnotes to Table 3.

### Data Synthesis

We grouped studies for each synthesis according to outcome and follow-up time point. Studies reporting data in a comparable format were included in random-effects meta-analyses when at least 2 comparable studies were available. We calculated RRs with 95% CIs for all outcomes based on available data from intention-to-treat study groups, deriving them from raw data or alternative effect measures, and presented pooled results as forest plots. Heterogeneity was assessed using the *I*^2^ statistic and interpreted in relation to the direction and consistency of effects across studies. For outcomes for which data quality or heterogeneity did not support meta-analysis, we reported results descriptively and interpreted them exploratorily. Prespecified exploratory subgroup analyses were performed by country income level (low- and middle-income countries [LMICs] versus high-income countries [HICs]). One included cluster RCT (Dehlendorf et al [22]) was randomized at the provider level (28 providers; mean cluster size of ≈27). Raw event counts per study arm were extracted from the published article. As the reported provider-level intracluster correlation coefficient was approximately 0, the corresponding design effect was ≈1.0, and no design-effect adjustment was applied. Sensitivity analyses examined the effect of publication year (before vs after 2015), which stratifies the approximately 20-year period during which telemedicine has been used for reproductive health, and low versus moderate or high RoB. Where meta-analysis was not feasible, we summarized findings narratively using incidence ranges. We conducted analyses using SPSS (version 30; IBM Corp) and Review Manager (RevMan 5, desktop version).

### Ethics Considerations

This review used only publicly available data and required no ethical approval. There was no funding source for this study. GPT-4o (OpenAI, May 2024) was used as a preliminary tool for data extraction of setting, population, intervention, and comparator characteristics from full-text articles; for the generation of a conceptual figure; and to support language editing. All AI-assisted data extraction outputs were verified and corrected against the source articles by reviewer MW and subsequently by ME. All analyses, interpretations, and conclusions were independently conducted by the authors. Outcomes related to potential harms were not prespecified, which we acknowledge as a limitation of the protocol.

The initial protocol was amended in the following ways before data extraction: outcomes were reorganized to specify a single primary outcome; the population eligibility criterion was clarified to restrict inclusion to women and girls of any age (including adolescents); men and couples were excluded; and subgroup and sensitivity analyses were added. The revised protocol also clarified our a priori intent to restrict the search to English-language publications.

### Patient and Public Involvement

Patients or members of the public were not involved in the design, conduct, reporting, or dissemination of this systematic review.

## Results

### Study Selection and Characteristics

**Figure 1:**
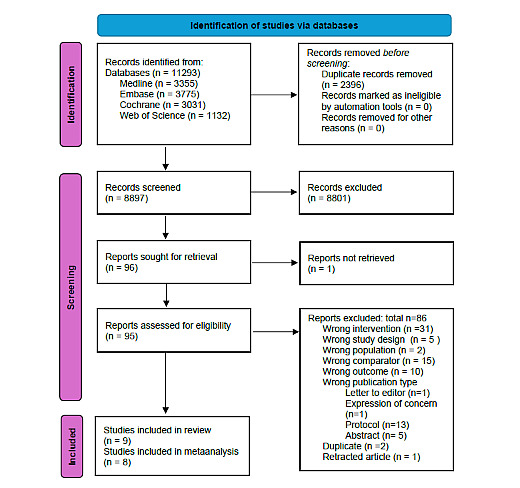
PRISMA (Preferred Reporting Items for Systematic Reviews and Meta-Analyses) flowchart.

From 8897 records screened, 8 individual RCTs and 1 cluster RCT met the inclusion criteria, involving 5353 participants from 5 HICs and 4 middle-income countries. The PRISMA flowchart illustrates the selection process (Figure 1), and Table 1 presents the study characteristics and sample sizes. Included studies were conducted in antenatal care [23,24], postabortion care [25-27], and routine contraceptive services [22,28-30]. Sample sizes ranged from 86 to 1475 participants.

All interventions fulfilled the 3 core components of our definition of TECC—telehealth delivery modality, interactivity, and tailoring—but the degree of interactivity and tailoring varied. Eight were hybrid models, delivered in addition to, before, during, or after an in-person clinical visit. Only one study [27] tested a standalone model that substituted for in-person care using telephone calls, but recruitment was terminated after only 125 of the 1222 (10.23%) planned enrollment due to changes in the comparator condition in response to the COVID-19 pandemic. The TECC modality and degree of interactivity ranged from potentially interactive voice [25,26] or SMS text messaging [23] with interaction on demand; asynchronous, self-guided computer- [28,29] or tablet-based modules [22,30]; to video or telephone calls delivered in real time [24,27]. Provider involvement in the TECC component, therefore, ranged from minimal to high. Tailoring also varied, ranging from SMS text or voice messages tailored to an already chosen method [23,25,26] to decision aids limited to life preferences and excluding medical criteria [29], comprehensive tailored decision aids [22,28,30], and real-time individualized assessments [24,27].

**Table 1:** Study characteristics.

Study^a^	Country (income classification)^b^	Setting	Population	Study design	Total sample size, n	Participants per arm, n
Reiss et al [25]	Bangladesh (lower middle income)	Public postmenstrual regulation services	Women aged 18-49 following menstrual regulation, with access to a personal mobile phone	RCT^c^ (2 arms)	969	Intervention: 485 and control: 484
Stephenson et al [29]	United Kingdom (high income)	Sexual and reproductive health clinics and online booking system	Women aged 15-30 with current or future contraceptive needs	RCT (2 arms)	927	Intervention: 464 and control: 463
Harrington et al [23]	Kenya (lower middle income)	Kenyan public hospitals (antenatal to postpartum)	Women and girls aged >14 years, pregnant ≥28 weeks’ gestation, and HIV negative, with daily phone access (partners optional)	RCT (2 arms)	260	Intervention: 130 and control: 130
Garbers et al [28]	United States (high income)	Urban family planning clinics	Women aged >16 years at risk of unintended pregnancy and attending family planning visits	RCT (3 arms)^d^	1475	Intervention: 985 and control: 490
Saglam Aksut and Inam [24]	Turkey (upper middle income)	Antenatal primary care	Pregnant women registering for antenatal care and using smartphones	RCT (2 arms)	86	Intervention: 43 and control: 43
Smith et al [26]	Cambodia (lower middle income)	Marie Stopes abortion clinics in peri-urban and rural settings	Women aged >17 years seeking abortion services, with access to a personal or predominantly private smartphone	RCT (2 arms)	500	Intervention: 249 and control: 251
Dehlendorf et al [22]	United States (high income)	Urban family planning centers, public health centers, college student health centers, and hospital outpatient clinics	Women aged 15-45 seeking contraception and not planning pregnancy within the next 7 months	Cluster RCT (2 arms)	Providers: 28; participants: 758	Providers—intervention: 15 and control: 13; participants—intervention: 407 and control: 351
Madden et al [30]	United States (high income)	Obstetrics/gynecology clinics at academic centers	Women aged 18-45 at risk of unintended pregnancy and planning to discuss contraception	RCT (2 arms)	253	Intervention: 167 and control: 86
Reynolds-Wright et al [27]	United Kingdom (high income)	Public abortion care center in Edinburgh	Pregnant individuals aged >16 years seeking medical abortion at home	RCT (2 arms)	125	Intervention: 63 and control: 62

^a^Studies are listed in the order in which the data were first extracted.

^b^Country income classes according to The World Bank classification.

^c^RCT: randomized controlled trial.

^d^Only 1 intervention arm met the inclusion criteria and is reported in this review.

**Figure 2:**
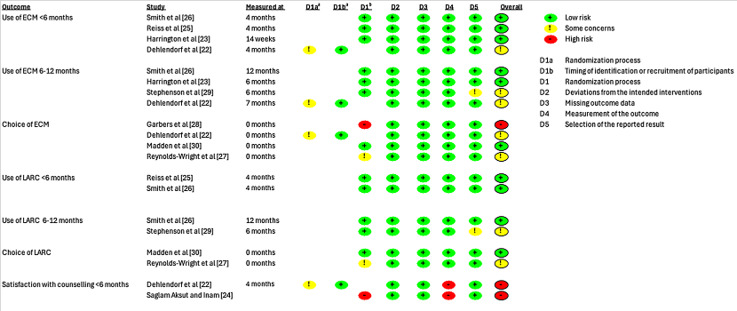
Studies grouped by outcome and Risk of Bias (RoB) related to outcome. ^a^: Cluster-randomized trial domain. ^b^: Individual randomized trial domain.

Comparators were standard in-person CC, with or without printed materials. In 1 trial [29], controls were offered delayed access to TECC after study completion. Table 2 outlines the intervention and comparator characteristics. Studies grouped by outcome and RoB assessment are presented in Figure 2 (also see [22-30]). Multimedia Appendix 5 details study outcomes and findings, and Multimedia Appendix 6 details how each intervention met the tailoring and interactivity criteria, as well as additional intervention characteristics that may have modified effects. GRADE-assessed certainty of evidence ranged from low to very low across outcomes. The GRADE assessment is presented in the summary of findings (Table 3), and a breakdown of each GRADE assessment is provided in Multimedia Appendix 7; 7 hybrid models and 1 standalone model contributed outcome data to the meta-analyses. One hybrid model contributed data for the outcome of satisfaction, which was reported descriptively [24].

**Table 2:** Intervention and comparator characteristics.

Study^a^	Relation to clinical care	Intervention	Timing and duration	Comparator
Reiss et al [25]	Hybrid/adjunct	Automated interactive voice messages delivered to mobile phones providing contraceptive method information and addressing concerns; messages tailored to the method chosen at menstrual regulation and updated if the method is switched; option to request callbacks for additional individual counseling	Delivered during the 4 months following in-person menstrual regulation; included 7 weekly messages and 4 fortnightly messages over 4 months	Routine in-person menstrual regulation counseling care without digital follow-up
Stephenson et al [29]	Hybrid/adjunct	Mobile-optimized website with videos, frequently asked questions, and a tailored decision aid generating 3 matched contraceptive methods based on user-reported preferences; designed to support informed choice before in-person counseling	Self-directed use before a clinical appointment	Routine in-person contraceptive counseling; website access offered after study follow-up
Harrington et al [23]	Hybrid/adjunct	SMS text messages tailored to user language and gestational age/postpartum stage, prompting user responses; nurses engaged in asynchronous 2-way communication; optional partner enrollment included	Weekly automated messages delivered from 28 weeks of gestation to 6 months postpartum	Routine in-person antenatal and postpartum care with no SMS text message follow-up
Garbers et al [28]	Hybrid/adjunct	Audio-assisted touchscreen decision aid eliciting user preferences and medical history, producing a traffic-light classification of contraceptive methods (green=most suitable; yellow=less effective or less suitable; and red=medically contraindicated methods); designed to support precounseling decision-making	Self-directed, 1-time use on a touchscreen laptop in the waiting room before a clinical appointment	Routine in-person contraceptive counseling and attention-matched control (demographic survey on laptop and generic printed handout)
Saglam Aksut and Inam [24]	Hybrid/adjunct	Video counseling calls using a structured handbook and visual demonstration of contraceptive methods before in-person visits; real-time tailored information provided with opportunity for user questions; partners included where possible	Provided in the third trimester of pregnancy as 2 sessions of approximately 50 minutes each, delivered 2 weeks apart	Routine in-person contraceptive counseling during antenatal visit at 24 weeks of gestation
Smith et al [26]	Hybrid/adjunct	Automated interactive voice messages providing tailored contraceptive information based on current method use; optional reminders for method adherence and option to request counseling callbacks	Initiated within 1 week after abortion and continued up to 3 months after abortion; fortnightly messages	Routine in-person postabortion care; no digital follow-up
Dehlendorf et al [22]	Hybrid/adjunct	Web-based interactive decision aid incorporating educational content, user preferences, reproductive life goals, and medical eligibility criteria; generates a personalized summary report with recommendations for shared decision-making during consultation	Self-directed, 1-time use on a tablet in the waiting room before a clinic visit	Routine in-person contraceptive counseling
Madden et al [30]	Hybrid/adjunct	Tablet-based interactive decision aid assessing user preferences, reproductive and medical history, prior contraceptive use, and preferences; generates top 3 tailored contraceptive recommendations and provides printed personalized information	Self-directed, 1-time use on a tablet in the waiting room before a clinic visit	Routine in-person contraceptive counseling with attention-matched control (demographic and reproductive survey on tablet plus generic handout)
Reynolds-Wright et al [27]	Standalone	Telephone counseling providing abortion-related counseling and postabortion contraceptive information; tailored real-time discussion during the call	Delivered at booking or another chosen time; replaced in-person counseling; followed by in-person ultrasound and medication provision; 1-time session	Routine in-person counseling

^a^Studies are listed in the order in which the data were first extracted.

### Risk of Bias

RoB assessments for each outcome are presented in Figure 2. Four studies [23,25,26,30] had low RoB across all domains and outcomes. The most frequent concerns related to the randomization process, including sequential allocation to control and intervention arms, imbalanced distribution of study groups across study sites, changes to the randomization procedure [28], and small but potentially meaningful baseline imbalances [22,24,27,28].

Stephenson et al [29] had moderate RoB in domain 5 (selection of the reported result) due to post hoc changes in study outcomes when the trial expanded from a feasibility study to an effectiveness trial without a prespecified analysis plan. We therefore judged this outcome to have moderate RoB. We classified the studies by Saglam Aksut and Inam [24] and Dehlendorf et al [22] as having high RoB for satisfaction with counseling outcomes because outcome measurement was conducted by study staff, creating a risk of social desirability bias.

### Use of ECMs

**Figure 3:**
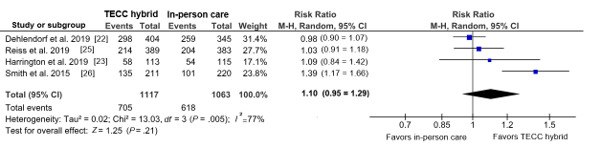
Forest plot of four studies comparing hybrid telehealth contraceptive counseling (TECC) with in-person care for effective contraceptive method use within 6 months of the intervention. M-H: Mantel–Haenszel method.

Four studies (n=2180) reported on our primary outcome, use of ECM less than 6 months after the intervention. The meta-analysis indicated a small positive but nonsignificant (*P*=.21) effect of TECC compared with standard care (SC; RR 1.10; 95% CI 0.95-1.29; *I*^2^=77%; Figure 3; see also [22,23,25,26]). The certainty of evidence was low, downgraded for inconsistency and imprecision (Table 3).

**Table 3:** Summary of findings and GRADE^a^.

Outcome and follow-up	Patients (studies), N	Relative effect, risk ratio (95% CI)	Absolute effects (95% CI)				Certainty		What happens
			Standard care	TECC^b^	Difference				
Use of ECM^c^ <6 months	2180 (4 RCTs^d^)^e^	1.10 (0.95-1.29)	582 per 1000	646 per 1000 (553-751)	64 more per 1000 (from 29 fewer to 169 more)	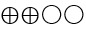 Low^f^^,^^g^		TECC may result in little to no difference in use of any effective contraceptive method at 0-6 months.	
Use of ECM at 6-12 months	2043 (4 RCTs)^e^	1.070 (1.002-1.130)	646 per 1000^e^	685 per 1000 (648-730)^e^	39 more per 1000 (from 1 more to 84 more)	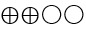 Low^h^^,^^i^		TECC may result in a slight increase in use of any effective contraceptive method at 6-12 months.	
Use of LARC^j^ <6 months	1203 (2 RCTs)	1.62	129 per 1000	210 per 1000	81 more per 1000	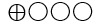 Very low^k^^,^^l^		The evidence is very uncertain about the effect of TECC on use of LARC at 0-6 months.	
Use of LARC 6-12 months	1042 (2 RCTs)^e^	Not estimable	N/A	N/A^m^	N/A	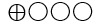 Very low^n^^,^^o^^,p^		The evidence is very uncertain about the effect of TECC on use of LARC at 6-12 months.	
Choice of ECM	2590 (4 RCTs)	1.07 (0.96-1.18)	564 per 1000	603 per 1000 (547-660)	39 more per 1000 (from 17 fewer to 96 more)	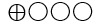 Very low^g^^,^^q^^,^^r^		The evidence is very uncertain about the effect of telemedicine on the choice of effective contraceptive methods.	
Choice of LARC	366 (2 RCTs)	Not estimable	N/A	N/A	N/A	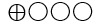 Very low^n^^,^^s^		The evidence is very uncertain about the effect of telemedicine on the choice of LARC.	
Satisfaction with counseling <6 months	749 (2 RCTs)	Not estimable	N/A	N/A	N/A	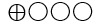 Very low^t^^,^^u^^,^^v^		The evidence is very uncertain about the effect of TECC on satisfaction with counseling up to 6 months.	
Use of ECM <6 months in LMIC^w^ or low RoB^x^	580 (2 RCTs)^e^	1.20 (1.03-1.30)	500 per 1000^e^	600 per 1000 (515-695)^e^	100 more per 1000 (from 15 more to 195 more)	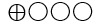 Very low^y^^,^^z^		The evidence is very uncertain about the effect of TECC on use of any effective contraceptive method at 0-6 months in LMICs.	

^a^GRADE: Grading of Recommendations Assessment, Development, and Evaluation.

^b^TECC: telehealth contraceptive counseling.

^c^ECM: effective contraceptive method.

^d^RCT: randomized controlled trial.

^e^Numbers calculated from percentages, rounded to whole numbers.

^f^Heterogeneity *I*^2^=0.77.

^g^Optimal information size adequate. Downgraded for imprecision because the effect estimate CI overlaps with no effect.

^h^Moderate RoB in 2/4 studies.

^i^Optimal information size is adequate, but the lower bound of the effect estimate CI (1002) fails to reliably exclude no effect.

^j^LARC: long-acting reversible contraception.

^k^Heterogeneity *I*^2^=0.96.

^l^Optimal information size not met. Very wide CI of the effect estimate. Lower bound below 1; fails to exclude no effect.

^m^N/A: not applicable.

^n^Moderate RoB in 1/2 studies

^o^Heterogeneity *I*^2^=0.87.

^p^Results not pooled. Smith et al [26] showed a very wide CI of effect estimate, whereas for Stephenson et al [29], the lower bound of the CI fails to exclude no effect.

^q^High and moderate RoB in 2/2 studies, respectively.

^r^Heterogeneity *I*^2^=0.53.

^s^Total sample sizes are small. The lower bound of the effect estimate CI fails to exclude no effect.

^t^High RoB in 2/2 studies

^u^Data cannot be pooled due to different measurement methods. One study showed no difference, and the other study suggested higher satisfaction in the intervention group.

^v^Data cannot be pooled. The lower bound of the effect estimate CI (Dehlendorf et al [22]) fails to exclude no effect.

^w^LMIC: low- and middle-income country.

^x^RoB: risk of bias.

^y^Heterogeneity *I*^2^=0.7.

^z^Wide CI of effect estimate. Lower bound below 1.

**Figure 4:**
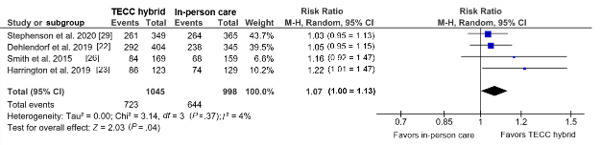
Forest plot of four studies comparing hybrid telehealth contraceptive counseling (TECC) with in-person care for effective contraceptive method use at 6-12 months after intrevention. M-H: Mantel–Haenszel method.

Four studies (n=2043) reported data on ECM use at 6-12 months after the intervention. The meta-analysis showed a small positive effect that reached statistical significance (*P*=.04), although the CI only narrowly excluded the null (RR 1.07; 95% CI 1.002-1.130; *I*^2^=4%; Figure 4; see also [22,23,26,29]). The certainty of evidence was low, downgraded for RoB and imprecision (Table 3).

**Figure 5:**
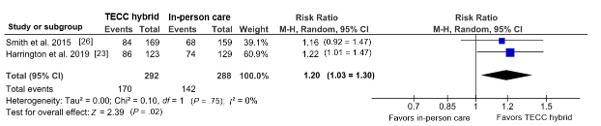
Forest plot of two low-risk-of-bias studies conducted in low- and middle-income countries, comparing hybrid telehealth contraceptive counseling (TECC) with in-person care for effective contraceptive method use at 6-12 months after intervention. M-H: Mantel–Haenszel method.

Our subgroup and sensitivity analyses assessed whether the effect of TECC on ECM choice and use varied by country income level, RoB, and publication period. Two subgroups overlapped: studies evaluating ECM use in LMICs also had low RoB. These analyses included a small number of studies, and the results should be interpreted with caution. Pooled results for ECM use at 6-12 months among studies conducted in LMICs with low RoB (n=580) showed that TECC significantly (*P*=.02) increased use (RR 1.20; 95% CI 1.03-1.30; *I*^2^=0%; Figure 5; see also [23,26]). The certainty of evidence was low, downgraded for serious imprecision (Table 3). We found no evidence of an effect on ECM choice or ECM use at 6-12 months in studies from HICs with moderate-to-high RoB, or on ECM use less than 6 months in LMICs. Comparisons by publication period (pre- versus post-2015) showed no differences in ECM choice or use. Subgroup analyses based on geographic region and ECM use less than 6 months in HICs were not possible because of the small number of studies.

### LARC Use

Two studies (n=1203) reported on LARC use at 0-6 months. Both studies were conducted in low-income settings in a postabortion context and used interactive voice messaging for TECC. However, statistical heterogeneity was high (*I*^2^=96%), and pooled estimation was not considered appropriate. Reiss et al [25] reported no significant difference between groups (RR 0.80; 95% CI 0.56-1.14), whereas Smith et al [26] found an effect in favor of the telehealth intervention (RR 3.35; 95% CI 2.07-5.40). The certainty of evidence was very low, downgraded for moderate RoB and serious imprecision (Table 3).

Two studies (n=1042) reported LARC use at 6-12 months (Smith et al [26] and Stephenson et al [29]). Statistical heterogeneity was very high (*I*^2^=87%) across 2 studies with different populations (postabortion care in Cambodia vs routine sexual health clinic attendees in the United Kingdom) and delivery modalities. Given these differences, pooled estimation was not considered appropriate. Smith et al [26] reported higher LARC use in the intervention group at 6 months compared with in-person care (RR 2.08; 95% CI 1.27-3.42), whereas Stephenson et al [29] reported no difference at 12 months (RR 0.98; 95% CI 0.79-1.22). The certainty of evidence was very low, downgraded for RoB, inconsistency, and serious imprecision (Table 3).

### Method Choice

**Figure 6:**
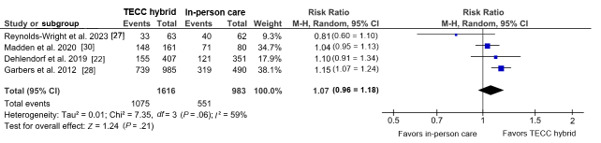
Forest plot of four studies comparing hybrid telehealth contraceptive counseling (TECC) with in-person care for choice of effective contraceptive method at the time of counseling. M-H: Mantel-Haenszel method.

Four studies (n=2599) assessed the choice of ECM immediately after the intervention. The meta-analysis showed no clear evidence (*P*=.21) of an effect of TECC compared with SC (RR 1.07; 95% CI 0.96-1.18; *I*^2^=59%; Figure 6; see also [22,27,28,30]). The certainty of evidence was very low, downgraded for RoB, inconsistency, and imprecision (Table 3).

Two small studies (n=366) assessed the choice of LARC: 1 was conducted in a postabortion setting in the United Kingdom and was terminated early, and the other was conducted in general gynecologic clinics in the United States. Pooled analysis was not considered appropriate because the total sample size did not meet the OIS threshold and clinical heterogeneity was high across study populations, interventions, and contexts. Neither study showed increased choice of LARC at the time of counseling after TECC compared with in-person care: Reynolds-Wright et al [27] (RR 0.77; 95% CI 0.30-1.93) and Madden et al [30] (RR 1.04; 95% CI 0.74-1.47). The certainty of evidence was very low, downgraded for RoB and imprecision (Table 3).

Subgroup and sensitivity analyses examining whether the effect of TECC on ECM choice varied by publication period (*P*=.52) or RoB (*P*=.55) showed no significant effects. Subgroup analyses of LARC choice could not be performed because of the small number of studies.

### Satisfaction With Counseling

Two studies (n=749) reported on satisfaction with counseling (Saglam Aksut and Inam [24] and Dehlendorf et al [22]). Madden et al [30] noted no difference in satisfaction but did not report data suitable for extraction. Meta-analysis was not possible because of differences in outcome measurement and reporting. Saglam Aksut and Inam [24] reported significantly higher satisfaction scores for TECC compared with SC (mean 174.86, SD 7.71 vs mean 154.63, SD 26.54; *P*<.001). However, both groups scored well above the predefined threshold for high satisfaction (142 on the 185-point scale used). Dehlendorf et al [22] found no difference in overall satisfaction between groups (odds ratio [OR] 1.11; 95% CI 0.74-1.67), but a higher proportion of TECC users assigned the highest quality ratings (266/404, 65.8% vs 198/345, 57.4%; OR 1.45; 95% CI 1.03-2.05), suggesting that TECC improved perceived counseling quality. The certainty of evidence was very low, downgraded for high RoB, inconsistency, and imprecision (Table 3).

### Method Switching

One study reported on method switching following TECC, but did not provide sufficient detail on the methods switched from and to; therefore, the outcome could not be analyzed [29].

### Adverse Events and Safety

Only 2 studies explicitly evaluated potential harms as prespecified outcomes. Reiss et al [25] found a statistically significant increase in self-reported physical intimate partner violence (IPV) in the intervention arm compared with the control arm after menstrual regulation (11% vs 7%; adjusted OR 1.97; 95% CI 1.12-3.46). Menstrual regulation is a pragmatic term used for abortion care in Bangladesh, where abortion is illegal. This finding was detectable only when closed questions naming specific acts of violence were used; the open adverse event question (Did anything happen to you as a result of being in this study?) yielded no reports of violence in either arm. Smith et al [26] found no evidence of adverse outcomes, including domestic abuse or road traffic accidents. Other studies did not prespecify or systematically report unintended consequences, representing a gap in the evidence.

### Heterogeneity and Publication Bias

We observed 2 forms of heterogeneity. Clinical heterogeneity was considerable, arising from differences in intervention design, including timing, delivery modality, provider involvement, and study setting. Studies were conducted across a range of care settings, including antenatal, postpartum, abortion, family planning, and general gynecologic clinics. Given the small number of studies available for each outcome, stratified analyses by clinical setting were not feasible. Although all interventions met our inclusion criteria for tailoring and interactivity, the extent of user engagement and integration with clinical care varied. Second, statistical heterogeneity was considerable for some outcomes (*I*^2^>85%), as reflected by the wide CIs around pooled effect estimates. The scarcity of positive effect sizes did not suggest publication bias; however, the small number of studies per outcome limited the ability to detect asymmetry, and imputation-based analyses were not conducted.

## Discussion

### Principal Findings

This review identified 9 RCTs comparing TECC models with in-person care, 8 of which were hybrid models, and the remaining 1 was a standalone model that was terminated early. We found no clear evidence of an effect of TECC on ECM use less than 6 months. We found a small, statistically significant increase in ECM use at 6-12 months (RR 1.07; 95% CI 1.002-1.130). However, this finding is fragile: the lower bound of the CI only narrowly excludes no effect, meaning that a small shift in events could render the result nonsignificant, and the certainty of evidence is low. Given that the pooled estimates for method use were derived from trials with heterogeneous interventions and low-certainty evidence, the small effect observed at 6-12 months should be interpreted with caution. For other outcomes, including choice of ECM, choice of LARC, LARC use at less than 6 months and 6-12 months, and satisfaction with counseling, the evidence was very uncertain compared with SC. Method switching was assessed in 1 trial [29] but was not reported in sufficient detail to permit analysis. Across outcomes, the certainty of evidence ranged from low to very low. Hybrid interventions dominated the included studies; therefore, no conclusions could be drawn regarding the effectiveness of standalone TECC models.

The included trials were designed to evaluate the superiority of TECC over standard in-person care, not its noninferiority. In contexts where geographic, financial, or structural barriers limit access to in-person CC, a telehealth alternative that achieves equivalent outcomes could still represent a clinically meaningful advance by enabling access to care that would otherwise be unavailable. The current evidence base cannot exclude the possibility that TECC is noninferior to standard in-person care; however, formally demonstrating noninferiority would require adequately powered noninferiority trials with prespecified margins.

### Comparison With Prior Work

In this review, we included only RCTs of tailored, interactive TECC, a definition that has not been used in any previous systematic review. Three previous systematic reviews, all published before 2020 and before the rapid expansion of telehealth during the COVID-19 pandemic, have assessed the effect of telehealth interventions on contraceptive use or choice [11,12,15].

Our primary outcome, ECM use, has been investigated in 1 previous review [11], which included 7 RCTs conducted in LMICs, 3 of which overlapped with our review [23,25,26]. Their meta-analysis showed no overall benefit of telehealth on ECM use compared with SC, but found a small effect in favor of telehealth in a subgroup analysis of 4 studies that evaluated ECM use as a primary outcome. Unlike our review, they included noninteractive interventions and excluded telephone-based interventions. Broader contraceptive use and continuation outcomes have been investigated in a review of telehealth decision aids [12], which included 11 RCTs, 4 of which overlapped with our review [22, 28-31]. Of the remaining 7 studies, 6 did not use SC comparators, and 1 intervention was not tailored. Their meta-analysis showed a small increase in use of any contraceptive method following telehealth contraceptive services compared with controls. Additionally, a recent cluster RCT among Latinx adolescents in the United States showed increased contraceptive uptake with a tailored TECC intervention, but the study was excluded from our review because not all controls received CC [32].

Contraceptive method choice has been evaluated in 1 review that included 5 RCTs, none of which were included in our review because they evaluated noninteractive interventions, such as video-based interventions, and defined their outcome as choice of any contraceptive method [15]. Consistent with our findings, their narrative synthesis showed that telehealth had no effect on method choice in 4 of the 5 trials.

Evidence on satisfaction with and acceptability of telehealth contraceptive services comes predominantly from observational studies conducted in the United States during the COVID-19 pandemic, which consistently report high acceptability, with users valuing convenience and rating counseling quality as comparable to in-person care [8-10,33]. However, a recent evidence map of telehealth sexual and reproductive health studies in high-income settings found that all observational and quasi-experimental studies demonstrated a very high susceptibility to confounding and selection bias, with many relying on convenience samples likely to overestimate acceptability and effectiveness [34]. These findings may also not reflect the experiences of populations with limited digital access or those who prefer in-person care. In LMICs, promising results regarding acceptability have been reported in pilot studies of telehealth communication for contraception among postabortion women in South Africa and among men and their spouses in Uganda [35,36].

### Strengths and Limitations

This review has several strengths, including adherence to a preregistered protocol, rigorous application of RoB 2 and GRADE, and an exclusive focus on RCTs of interactive and tailored interventions to examine the causal effect of the telehealth counseling modality compared with in-person care. One limitation is the restriction to English-language publications, which may have introduced language bias and excluded relevant non-English-language trials. A further limitation is the exclusion of observational evidence. Prespecification of RCT-only inclusion strengthens causal inference regarding the effect of the telehealth counseling modality but constrains external validity and excludes a substantial body of real-world implementation data that have grown since the expansion of telehealth during the COVID-19 pandemic. Observational studies provide important insights into who accesses telehealth contraceptive services and how these services are experienced, but they are less suited to answering the specific effectiveness question addressed in this review because patients who select telehealth differ systematically from those who attend in-person care with respect to age, language, insurance status, income, and digital literacy. In addition, observational designs in this field have been shown to be highly susceptible to confounding and selection bias [34,37].

We used the standard DerSimonian-Laird random-effects approach in our meta-analysis. The Hartung-Knapp-Sidik-Jonkman method should be considered in future systematic reviews, particularly when the risk of spurious positive findings (type I error) is greater than it was in our analysis. The evidence has important limitations. We did not contact the study authors for additional or clarifying information. Interventions differed widely in content, delivery, timing, degree of provider involvement, and clinical context, potentially contributing to substantial heterogeneity in the results. Outcomes were often self-reported, and group allocation was unblinded, increasing RoB for subjective outcomes such as satisfaction. Some studies were excluded because outcome data did not distinguish between any contraceptive method and ECM as defined in our review. The small number of trials limited subgroup analyses and reduced power to explore sources of heterogeneity through sensitivity analyses. In line with Cochrane guidance, we did not present funnel plots to assess publication bias because fewer than 10 studies were included. We do not suspect publication bias, as most trials showed no effect in favor of the intervention.

Despite our focus on comprehensive TECC, tailoring and interactivity were only partially implemented in most included trials, highlighting a gap in TECC intervention design that may have limited effectiveness. Our review did not assess which components of TECC were most influential in determining outcomes. Previous reviews have attempted to identify the most effective features and have suggested that the incorporation of behavior change techniques and motivational interviewing may increase effectiveness [11,13,14]. However, none were able to determine which specific components were most effective.

The included trials were conducted across antenatal, postabortion, and routine contraceptive care settings, and in both high- and middle-income countries, supporting the generalizability of our findings to some extent. However, important gaps remain: no trial was conducted in a low-income country, and adolescents were represented in only 2 trials [22,28]. These gaps limit the generalizability of the findings to populations with a high unmet need for contraception. Furthermore, only 1 trial [27], which was terminated early, evaluated standalone TECC, highlighting the evidence gap regarding standalone TECC.

Safety outcomes were seldom assessed. One trial reported increased IPV following a mobile-based intervention in Bangladesh [25]. The intervention explicitly encouraged women to discuss contraception with their partners and included content aimed at increasing partner engagement in contraceptive decision-making. Mobile-based interventions that generate records or notifications may inadvertently increase the risk of IPV. This finding underscores the critical importance of prospective safety monitoring, harm-informed design, and safeguard protocols in all TECC evaluations, particularly in settings with a high prevalence of IPV. Although this finding may reflect context-specific factors related to postmenstrual regulation services in a setting with a high prevalence of IPV, it should not be dismissed as an isolated observation. Future TECC trials should consider incorporating routine baseline IPV risk screening, prespecified adverse event monitoring protocols, and context-sensitive content design that avoids prompting partner communication when doing so may increase risk.

Observational studies conducted in the United States have shown that food and housing insecurity, language barriers, lack of insurance, and race are associated with reduced access to telehealth contraceptive care [37,38]. Equity dimensions were not systematically addressed in the included trials, underscoring the need for equity-focused implementation and evaluation in diverse and underserved populations, including individuals with low literacy and limited digital access.

### Implications

Although the evidence on adjunct TECC is inconclusive, it does not appear to substantially improve use, method choice, or satisfaction in settings where in-person counseling is already available. Our review assessed hybrid TECC in comparison with in-person counseling, which is not available in all contexts. If TECC were noninferior to SC in whatever form that takes, it may offer benefits in settings with limited trained providers, geographical or social barriers to care, or in overburdened health systems. In such settings, even modest effects might translate into population-level impact. The effectiveness of standalone TECC remains unknown, and its clinical relevance would depend on demonstrating that TECC is no less effective, safe, or acceptable than SC.

### Future Research

Future research should prioritize adequately powered evaluations of standalone TECC, including low-income settings and adolescents, and assess long-term outcomes such as continuation, switching, and unintended pregnancy. Noninferiority designs could establish clinical relevance in settings with limited access to in-person care. Mixed-methods approaches are needed to capture user experiences, equity implications, and unintended harms. Standardized and validated measures of contraceptive use, satisfaction, and decision quality would improve comparability. Complementary real-world effectiveness evidence could be generated through rigorous quasi-experimental designs, such as propensity score–matched cohort studies in routine care populations and interrupted time-series analyses that treat system-level changes, such as the COVID-19 telehealth expansion, as natural experiments. The rapid growth of online contraceptive platforms and AI-driven communication requires rigorous evaluation of their effectiveness, safety, and implementation. Evidence is needed to guide decision makers in integrating TECC into routine contraceptive services amid ongoing digital health system transformations.

### Conclusions

Current evidence suggests, with low certainty, that adjunct TECC delivered alongside in-person care shows little to no effect on contraceptive use compared with in-person care. For method choice, LARC uptake, and satisfaction compared with in-person care, the evidence was very uncertain. The effectiveness of standalone TECC remains unknown. High acceptability and scalability warrant further rigorous and equity-focused evaluations to inform integration into routine contraceptive services.

### Data Sharing

All data used in this article are from publicly available published sources. Extracted data are provided in the article and its multimedia appendices. Additional outcome data, risk-of-bias assessments, and statistical calculations for meta-analyses will be made available on reasonable request to the corresponding author (ME). The PROSPERO protocol (CRD42023404402) is publicly available.

## Appendix

Multimedia Appendix 1 PRISMA (Preferred Reporting Items for Systematic Reviews and Meta-Analyses) checklist.

Multimedia Appendix 2 Search strategies.

Multimedia Appendix 3 Artificial intelligence–generated conceptual framework of telehealth contraceptive counseling (TECC). The framework includes 3 core domains: (1) telehealth delivery via a user interface on a phone, laptop, or tablet; (2) interactive, bidirectional communication; and (3) personalization based on users’ preferences, reproductive goals, or medical eligibility. Additional characteristics may vary by degree of automation and by whether communication is delivered and responded to in real time or asynchronously. TECC may be integrated before, during, or after clinical visits and may serve either as a standalone substitute for, or a hybrid adjunct to, in-person counseling.

Multimedia Appendix 4 Records excluded at full-text review with reasons.

Multimedia Appendix 5 Outcomes and findings.

Multimedia Appendix 6 Characteristics of study interventions categorized according to the 3 domains of our telehealth contraceptive counseling definition: modality, tailoring, and interactivity.

Multimedia Appendix 7 Detailed breakdown of GRADE (Grading of Recommendations Assessment, Development, and Evaluation) assessment.

## Abbreviations

CC: contraceptive counseling
ECM: effective contraceptive method
GRADE: Grading of Recommendations Assessment, Development, and Evaluation
HIC: high-income country
IPV: intimate partner violence
LARC: long-acting reversible contraception
LMIC: low- and middle-income country
MeSH: Medical Subject Headings
OIS: optimal information size
OR: odds ratio
PRISMA: Preferred Reporting Items for Systematic Reviews and Meta-Analyses
PROSPERO: International Prospective Register of Systematic Reviews
RCT: randomized controlled trial
RoB: risk of bias
RR: relative risk
SC: standard care
TECC: telehealth contraceptive counseling
