# Supplementary material for: Tailored and Interactive Mobile Telehealth Contraceptive Counseling Compared With In-Person Care: Systematic Review and Meta-Analysis of Randomized Controlled Trials
Source: JMIR Mhealth Uhealth. 2026 Jul 16;14:e88887. doi: 10.2196/88887 (PMC13424753; doi:10.2196/88887)
Supplement: Multimedia Appendix 2 [file mhealth_v14i1e88887_app2.docx]

Documentation of search strategies

University Library search consultation group

Date: 2023-02-10 updated 2024-10-30 updated 2025-10-15

Topic/research question: How effective are telehealth interventions for contraceptive counseling?

Name of researcher(s): Maja Weinryb & Margit Endler, Institutionen för kvinnors och barns hälsa

Librarian(s): Lovisa Liljegren & Emma-Lotta Säätelä

Databases:

1. Medline (Ovid)
2. Web of Science (Clarivate)
3. Embase (embase.com)
4. Cochrane Library (Wiley)

Total number of hits:

- Before deduplication: 11,293
- After deduplication: 8,897

Search strategy:

A literature search was performed in the following databases: Medline, Web of Science, Embase and Cochrane Library. The last search was conducted 2023-02-10.

The search strategy was developed in Medline (Ovid) in collaboration with librarians at the Karolinska Institutet University Library. For each search concept Medical Subject Headings (MeSH-terms) and free text terms were identified. The search was then translated, in part using Polyglot Search Translator (1), into the other databases.

No language restriction was applied.

Databases were searched from inception.

The strategies were peer reviewed by another librarian prior to execution.

De-duplication was done using the method described by Bramer et al (2). One final, extra step was added to compare DOIs.

After the original search was performed on 2023-02-10, the search was last updated on 2025-10-15 by rerunning the searches and deduplicating against the previous results using Covidence (3).

References

1. Clark JM, Sanders S, Carter M, Honeyman D, Cleo G, Auld Y, Booth D, Condron P, Dalais C, Bateup S, Linthwaite B, May N, Munn J, Ramsay L, Rickett K, Rutter C, Smith A, Sondergeld P, Wallin M, Jones M, Beller E. (2020) Improving the translation of search strategies using the Polyglot Search Translator: a randomized controlled trial. *Journal of the Medical Library Association: JMLA*. 108(2):195-207. doi: 10.5195/jmla.2020.834.

2. Bramer, W. M., Giustini, D., de Jonge, G. B., Holland, L., & Bekhuis, T. (2016). De-duplication of database search results for systematic reviews in EndNote. *Journal of the Medical Library Association: JMLA*, 104(3), 240-243. doi: 10.3163/1536-5050.104.3.014

3. Covidence systematic review software, Veritas Health Innovation, Melbourne, Australia. Available at [www.covidence.org](http://www.covidence.org).

Reports assessed for eligibility

(n = )

**Identification of studies via databases and registers**

**Identification of studies via other methods**

Records removed *before screening*:

Duplicate records removed (n = 2396)

Records marked as ineligible by automation tools (n = )

Records removed for other reasons (n = )

Records identified from*:

Databases (n = 11,293

Medline n = 3355

Embase n = 3775

Cochrane n = 3031

Web of Science n = 1132)

Registers (n = )

Records screened

(n = 8897)

Records excluded**

(n = )

Reports sought for retrieval

(n = )

Reports sought for retrieval

(n = )

Reports not retrieved

(n = )

**Screening**

Reports excluded:

Reason 1 (n = )

Reason 2 (n = )

Reason 3 (n = )

etc.

Reports assessed for eligibility

(n = )

Reports excluded:

Reason 1 (n = )

Reason 2 (n = )

Reason 3 (n = )

etc.

Records identified from:

Websites (n = )

Organisations (n = )

Citation searching (n = )

etc.

**Identification**

Reports not retrieved

(n = )

Studies included in review

(n = )

Reports of included studies

(n = )

**Included**

*Consider, if feasible to do so, reporting the number of records identified from each database or register searched (rather than the total number across all databases/registers).

**If automation tools were used, indicate how many records were excluded by a human and how many were excluded by automation tools.

*From:*  Page MJ, McKenzie JE, Bossuyt PM, Boutron I, Hoffmann TC, Mulrow CD, et al. The PRISMA 2020 statement: an updated guideline for reporting systematic reviews. BMJ 2021;372:n71. doi: 10.1136/bmj.n71. For more information, visit: <http://www.prisma-statement.org/>

1. Medline

| Interface: Ovid MEDLINE(R) ALL  Date of Search: 2025-10-15  Number of hits: 3355  Comment: In Ovid, two or more words are automatically searched as phrases; i.e. no quotation marks are needed | Field labels   - exp/ = exploded MeSH term - / = non exploded MeSH term - .ti,ab,kf. = title, abstract and author keywords - adjx = within x words, regardless of order - * = truncation of word for alternate endings |
| --- | --- |
| Ovid MEDLINE(R) ALL <1946 to October 14, 2025>   \| 1 \| exp Contraception/ \| 31020 \| \| --- \| --- \| --- \| \| 2 \| Family Planning Services/ \| 27300 \| \| 3 \| exp Reproductive Behavior/ \| 10980 \| \| 4 \| Pregnancy, Unplanned/ \| 2715 \| \| 5 \| Pregnancy, Unwanted/ \| 2757 \| \| 6 \| Preconception Care/ \| 2957 \| \| 7 \| exp Contraceptive agents/ \| 81193 \| \| 8 \| (family planning or planned pregnan* or contracept* or fertility control or unplanned pregnan* or unintended pregnan* or unwanted pregnan* or birth spacing or birth interval* or preconception or reproductive behavior).ti,ab,kf. \| 122446 \| \| 9 \| (childbearing or childless* or Coitus Interruptus or Periodic Abstinence or Ovulation Inhibition or Ovulation Suppression or Sterilization).ti,ab,kf. \| 49247 \| \| 10 \| or/1-9 \| 217589 \| \| 11 \| exp Cell phone/ \| 26717 \| \| 12 \| exp Computers/ \| 90519 \| \| 13 \| Decision Support Techniques/ \| 23881 \| \| 14 \| Internet/ \| 87007 \| \| 15 \| Internet-Based Intervention/ \| 1847 \| \| 16 \| Mobile Applications/ \| 15847 \| \| 17 \| exp Remote Consultation/ \| 6245 \| \| 18 \| Social Media/ \| 20702 \| \| 19 \| Therapy, Computer-Assisted/ \| 7062 \| \| 20 \| Telemedicine/ \| 46394 \| \| 21 \| (econsultation* or ecounseling or e health* or ehealth* or e* prescri* or e therapies or e therapy or etherap* or e visit* or evisit* or m health or mhealth or tele health* or telehealth* or teleconsultation* or tele consultation* or tele medicine or telemedicine or telerehabilitation or tele rehabilitation).ti,ab,kf. \| 85482 \| \| 22 \| (remote* adj3 (advice or coaching or counsel* or consult* or intervention* or guidance* or rehabilitat* or support or tailor* or therap* or treat*)).ti,ab,kf. \| 7639 \| \| 23 \| (distance adj3 (advice or coaching or counsel* or consult* or intervention* or guidance* or rehabilitat* or support or tailor* or therap* or treat*)).ti,ab,kf. \| 2742 \| \| 24 \| (electronic* adj3 (advice or coaching or counsel* or consult* or intervention* or guidance* or rehabilitat* or support or tailor* or therap* or treat*)).ti,ab,kf. \| 6822 \| \| 25 \| (app or apps or chat* or cellphone* or computer* or cyber or digital or e mail or email or internet* or mobile application* or online or phone* or smartphone* or sms or social media or technolog* or telephone* or text messag* or video* or virtual or web).ti,ab,kf. \| 2310655 \| \| 26 \| (twitter or Instagram or facebook or tiktop or whatsapp or messenger).ti,ab,kf. \| 96777 \| \| 27 \| or/11-26 \| 2518874 \| \| 28 \| randomized controlled trial.pt. \| 647555 \| \| 29 \| controlled clinical trial.pt. \| 95743 \| \| 30 \| randomized.ab. \| 712989 \| \| 31 \| placebo.ab. \| 262801 \| \| 32 \| drug therapy.fs. \| 2860945 \| \| 33 \| randomly.ab. \| 471060 \| \| 34 \| trial.ab. \| 779322 \| \| 35 \| groups.ab. \| 2928565 \| \| 36 \| or/28-35 \| 6432893 \| \| 37 \| 36 and 27 and 10 \| 3481 \| \| 38 \| Limit 37 to English \| 3355 \| | |

2. Embase

| Interface: embase.com  Date of Search: 2025-10-15  Number of hits: 3775  Comment: Emtree is the controlled vocabulary in Embase | Field labels   - /exp = exploded Emtree term - /de = non exploded Emtree term - ti,ab,kw = title, abstract and author keywords - tt = original non-english title - NEAR/x = within x words, regardless of order - * = truncation of word for alternate endings |
| --- | --- |
| \| No. \| Query \| Results \| \| --- \| --- \| --- \| \| #33 \| #30 NOT #31 AND [english]/lim \| 3775 \| \| #32 \| #30 NOT #31 \| 3895 \| \| #31 \| #11 AND #28 AND #29 AND ('conference abstract'/it OR 'conference paper'/it OR 'conference review'/it OR 'clinical trial'/it) \| 4004 \| \| #30 \| #11 AND #28 AND #29 \| 7899 \| \| #29 \| ('randomized controlled trial'/de OR 'controlled clinical trial'/de OR random*:ti,ab,tt OR 'randomization'/de OR 'intermethod comparison'/de OR placebo:ti,ab,tt OR compare:ti,tt OR compared:ti,tt OR comparison:ti,tt OR ((evaluated:ab OR evaluate:ab OR evaluating:ab OR assessed:ab OR assess:ab) AND (compare:ab OR compared:ab OR comparing:ab OR comparison:ab)) OR ((open NEXT/1 label):ti,ab,tt) OR (((double OR single OR doubly OR singly) NEXT/1 (blind OR blinded OR blindly)):ti,ab,tt) OR 'double blind procedure'/de OR ((parallel NEXT/1 group*):ti,ab,tt) OR crossover:ti,ab,tt OR 'cross over':ti,ab,tt OR (((assign* OR match OR matched OR allocation) NEAR/6 (alternate OR group OR groups OR intervention OR interventions OR patient OR patients OR subject OR subjects OR participant OR participants)):ti,ab,tt) OR assigned:ti,ab,tt OR allocated:ti,ab,tt OR ((controlled NEAR/8 (study OR design OR trial)):ti,ab,tt) OR volunteer:ti,ab,tt OR volunteers:ti,ab,tt OR 'human experiment'/de OR trial:ti,tt) NOT (((random* NEXT/1 sampl* NEAR/8 ('cross section*' OR questionnaire* OR survey OR surveys OR database OR databases)):ti,ab,tt) NOT ('comparative study'/de OR 'controlled study'/de OR 'randomised controlled':ti,ab,tt OR 'randomized controlled':ti,ab,tt OR 'randomly assigned':ti,ab,tt) OR ('cross‐sectional study' NOT ('randomized controlled trial'/de OR 'controlled clinical study'/de OR 'controlled study'/de OR 'randomised controlled':ti,ab,tt OR 'randomized controlled':ti,ab,tt OR 'control group':ti,ab,tt OR 'control groups':ti,ab,tt)) OR ('case control*':ti,ab,tt AND random*:ti,ab,tt NOT ('randomised controlled':ti,ab,tt OR 'randomized controlled':ti,ab,tt)) OR ('systematic review':ti,tt NOT (trial:ti,tt OR study:ti,tt)) OR (nonrandom*:ti,ab,tt NOT random*:ti,ab,tt) OR 'random field*':ti,ab,tt OR (('random cluster' NEAR/4 sampl*):ti,ab,tt) OR (review:ab AND review:it NOT trial:ti,tt) OR ('we searched':ab AND (review:ti,tt OR review:it)) OR 'update review':ab OR ((databases NEAR/5 searched):ab) OR ((rat:ti,tt OR rats:ti,tt OR mouse:ti,tt OR mice:ti,tt OR swine:ti,tt OR porcine:ti,tt OR murine:ti,tt OR sheep:ti,tt OR lambs:ti,tt OR pigs:ti,tt OR piglets:ti,tt OR rabbit:ti,tt OR rabbits:ti,tt OR cat:ti,tt OR cats:ti,tt OR dog:ti,tt OR dogs:ti,tt OR cattle:ti,tt OR bovine:ti,tt OR monkey:ti,tt OR monkeys:ti,tt OR trout:ti,tt OR marmoset*:ti,tt) AND 'animal experiment'/de) OR ('animal experiment'/de NOT ('human experiment'/de OR 'human'/de))) \| 6729577 \| \| #28 \| #12 OR #13 OR #14 OR #15 OR #16 OR #17 OR #18 OR #19 OR #20 OR #21 OR #22 OR #23 OR #24 OR #25 OR #26 OR #27 \| 3393555 \| \| #27 \| twitter:ti,ab,kw OR instagram:ti,ab,kw OR facebook:ti,ab,kw OR tiktok:ti,ab,kw OR whatsapp:ti,ab,kw OR messenger:ti,ab,kw \| 112445 \| \| #26 \| app:ti,ab,kw OR apps:ti,ab,kw OR chat*:ti,ab,kw OR cellphone*:ti,ab,kw OR computer*:ti,ab,kw OR cyber:ti,ab,kw OR digital:ti,ab,kw OR 'e mail':ti,ab,kw OR email:ti,ab,kw OR internet*:ti,ab,kw OR 'mobile application*':ti,ab,kw OR online:ti,ab,kw OR phone*:ti,ab,kw OR smartphone*:ti,ab,kw OR sms:ti,ab,kw OR 'social media':ti,ab,kw OR technolog*:ti,ab,kw OR telephone*:ti,ab,kw OR 'text messag*':ti,ab,kw OR video*:ti,ab,kw OR virtual:ti,ab,kw OR web:ti,ab,kw \| 3081063 \| \| #25 \| (electronic* NEAR/3 (advice OR coaching OR counsel* OR consult* OR intervention* OR guid* OR rehabilitat* OR 'self guid*' OR support OR tailor* OR therap* OR treat*)):ti,ab,kw \| 12317 \| \| #24 \| (distance NEAR/3 (advice OR coaching OR counsel* OR consult* OR intervention* OR guid* OR rehabilitat* OR 'self guid*' OR support OR tailor* OR therap* OR treat*)):ti,ab,kw \| 4691 \| \| #23 \| (remote* NEAR/3 (advice OR coaching OR counsel* OR consult* OR intervention* OR guid* OR rehabilitat* OR 'self guid*' OR support OR tailor* OR therap* OR treat*)):ti,ab,kw \| 12009 \| \| #22 \| econsultation*:ti,ab,kw OR ecounseling:ti,ab,kw OR 'e health*':ti,ab,kw OR ehealth*:ti,ab,kw OR 'e* prescri*':ti,ab,kw OR 'e therapies':ti,ab,kw OR 'e therapy':ti,ab,kw OR etherap*:ti,ab,kw OR 'e visit*':ti,ab,kw OR evisit*:ti,ab,kw OR 'm health':ti,ab,kw OR mhealth:ti,ab,kw OR 'tele health*':ti,ab,kw OR telehealth*:ti,ab,kw OR teleconsultation*:ti,ab,kw OR 'tele consultation*':ti,ab,kw OR 'tele medicine':ti,ab,kw OR telemedicine:ti,ab,kw OR telerehabilitation:ti,ab,kw OR 'tele rehabilitation':ti,ab,kw \| 119479 \| \| #21 \| 'telemedicine'/de \| 57191 \| \| #20 \| 'therapy, computer-assisted'/de \| 4966 \| \| #19 \| 'social media'/de \| 72321 \| \| #18 \| 'teleconsultation'/exp \| 19143 \| \| #17 \| 'mobile application'/exp \| 37896 \| \| #16 \| 'web-based intervention'/de \| 5764 \| \| #15 \| 'internet'/de \| 135750 \| \| #14 \| 'decision support system'/de \| 31780 \| \| #13 \| 'computer'/exp \| 200192 \| \| #12 \| 'mobile phone'/exp \| 67488 \| \| #11 \| #1 OR #2 OR #3 OR #4 OR #5 OR #6 OR #7 OR #8 OR #9 OR #10 \| 490306 \| \| #10 \| childbearing:ti,ab,kw OR childlessness:ti,ab,kw OR 'coitus interruptus':ti,ab,kw OR 'periodic abstinence':ti,ab,kw OR 'ovulation inhibition':ti,ab,kw OR 'ovulation suppression':ti,ab,kw OR sterilization:ti,ab,kw \| 61179 \| \| #9 \| 'family planning':ti,ab,kw OR 'planned pregnan*':ti,ab,kw OR contracept*:ti,ab,kw OR 'fertility control':ti,ab,kw OR 'unplanned pregnan*':ti,ab,kw OR 'unintended pregnan*':ti,ab,kw OR 'unwanted pregnan*':ti,ab,kw OR 'birth spacing':ti,ab,kw OR 'birth interval*':ti,ab,kw OR preconception:ti,ab,kw OR 'reproductive behavior':ti,ab,kw \| 145238 \| \| #8 \| 'contraceptive agent'/exp \| 192088 \| \| #7 \| 'prepregnancy care'/de \| 3827 \| \| #6 \| 'pregnancy, unwanted'/de \| 4388 \| \| #5 \| 'pregnancy, unplanned'/de \| 8474 \| \| #4 \| 'contraceptive behavior'/exp \| 22693 \| \| #3 \| 'reproductive behavior'/de \| 2548 \| \| #2 \| 'family planning'/exp \| 46991 \| \| #1 \| 'contraception'/exp \| 212054 \| | |

3. Cochrane Library

| Interface: Wiley  Date of Search: 2025-10-15  Number of hits: 3031 | Field labels   - ti,ab,kw = title, abstract and author keywords - NEAR/x = within x words, regardless of order - * = truncation of word for alternate endings |
| --- | --- |
| \| ID \| Search \| Hits \| \| --- \| --- \| --- \| \| #1 \| [mh Contraception] \| 832 \| \| #2 \| [mh ^"Family Planning Services"] \| 440 \| \| #3 \| [mh "Reproductive Behavior"] \| 373 \| \| #4 \| [mh ^"Pregnancy, Unplanned"] \| 145 \| \| #5 \| [mh ^"Pregnancy, Unwanted"] \| 65 \| \| #6 \| [mh ^"Preconception Care"] \| 198 \| \| #7 \| [mh "Contraceptive agents"] \| 3508 \| \| #8 \| ("family planning":ti,ab,kw OR ("planned" NEXT pregnan*):ti,ab,kw OR contracept*:ti,ab,kw OR "fertility control":ti,ab,kw OR ("unplanned" NEXT pregnan*):ti,ab,kw OR ("unintended" NEXT pregnan*):ti,ab,kw OR ("unwanted" NEXT pregnan*):ti,ab,kw OR "birth spacing":ti,ab,kw OR ("birth" NEXT interval*):ti,ab,kw OR preconception:ti,ab,kw OR "reproductive behavior":ti,ab,kw) \| 21286 \| \| #9 \| (childbearing:ti,ab,kw OR childless*:ti,ab,kw OR "Coitus Interruptus":ti,ab,kw OR "Periodic Abstinence":ti,ab,kw OR "Ovulation Inhibition":ti,ab,kw OR "Ovulation Suppression":ti,ab,kw OR Sterilization:ti,ab,kw) \| 11477 \| \| #10 \| #1 OR #2 OR #3 OR #4 OR #5 OR #6 OR #7 OR #8 OR #9 \| 27147 \| \| #11 \| [mh "Cell phone"] \| 3817 \| \| #12 \| [mh Computers] \| 3136 \| \| #13 \| [mh ^"Decision Support Techniques"] \| 1486 \| \| #14 \| [mh ^Internet] \| 5647 \| \| #15 \| [mh ^"Internet-Based Intervention"] \| 996 \| \| #16 \| [mh ^"Mobile Applications"] \| 2629 \| \| #17 \| [mh "Remote Consultation"] \| 487 \| \| #18 \| [mh ^"Social Media"] \| 662 \| \| #19 \| [mh ^"Therapy, Computer-Assisted"] \| 1571 \| \| #20 \| [mh ^Telemedicine] \| 4775 \| \| #21 \| (econsultation*:ti,ab,kw OR ecounseling:ti,ab,kw OR ("e" NEXT health*):ti,ab,kw OR ehealth*:ti,ab,kw OR (e* NEXT prescri*):ti,ab,kw OR "e therapies":ti,ab,kw OR "e therapy":ti,ab,kw OR etherap*:ti,ab,kw OR ("e" NEXT visit*):ti,ab,kw OR evisit*:ti,ab,kw OR "m health":ti,ab,kw OR mhealth:ti,ab,kw OR ("tele" NEXT health*):ti,ab,kw OR telehealth*:ti,ab,kw OR teleconsultation*:ti,ab,kw OR ("tele" NEXT consultation*):ti,ab,kw OR "tele medicine":ti,ab,kw OR telemedicine:ti,ab,kw OR telerehabilitation:ti,ab,kw OR "tele rehabilitation":ti,ab,kw) \| 20530 \| \| #22 \| (remote*:ti,ab,kw NEAR/3 (advice:ti,ab,kw OR coaching:ti,ab,kw OR counsel*:ti,ab,kw OR consult*:ti,ab,kw OR intervention*:ti,ab,kw OR guidance*:ti,ab,kw OR rehabilitat*:ti,ab,kw OR support:ti,ab,kw OR tailor*:ti,ab,kw OR therap*:ti,ab,kw OR treat*:ti,ab,kw)) \| 3139 \| \| #23 \| (distance:ti,ab,kw NEAR/3 (advice:ti,ab,kw OR coaching:ti,ab,kw OR counsel*:ti,ab,kw OR consult*:ti,ab,kw OR intervention*:ti,ab,kw OR guidance*:ti,ab,kw OR rehabilitat*:ti,ab,kw OR support:ti,ab,kw OR tailor*:ti,ab,kw OR therap*:ti,ab,kw OR treat*:ti,ab,kw)) \| 822 \| \| #24 \| (electronic*:ti,ab,kw NEAR/3 (advice:ti,ab,kw OR coaching:ti,ab,kw OR counsel*:ti,ab,kw OR consult*:ti,ab,kw OR intervention*:ti,ab,kw OR guidance*:ti,ab,kw OR rehabilitat*:ti,ab,kw OR support:ti,ab,kw OR tailor*:ti,ab,kw OR therap*:ti,ab,kw OR treat*:ti,ab,kw)) \| 2541 \| \| #25 \| (app:ti,ab,kw OR apps:ti,ab,kw OR chat*:ti,ab,kw OR cellphone*:ti,ab,kw OR computer*:ti,ab,kw OR cyber:ti,ab,kw OR digital:ti,ab,kw OR "e mail":ti,ab,kw OR email:ti,ab,kw OR internet*:ti,ab,kw OR ("mobile" NEXT application*):ti,ab,kw OR online:ti,ab,kw OR phone*:ti,ab,kw OR smartphone*:ti,ab,kw OR sms:ti,ab,kw OR "social media":ti,ab,kw OR technolog*:ti,ab,kw OR telephone*:ti,ab,kw OR ("text" NEXT messag*):ti,ab,kw OR video*:ti,ab,kw OR virtual:ti,ab,kw OR web:ti,ab,kw) \| 246093 \| \| #26 \| (twitter:ti,ab,kw OR Instagram:ti,ab,kw OR facebook:ti,ab,kw OR tiktop:ti,ab,kw OR whatsapp:ti,ab,kw OR messenger:ti,ab,kw) \| 5397 \| \| #27 \| #11 OR #12 OR #13 OR #14 OR #15 OR #16 OR #17 OR #18 OR #19 OR #20 OR #21 OR #22 OR #23 OR #24 OR #25 OR #26 \| 259381 \| \| #28 \| #27 AND #10 in Trials \| 3100 \| \| #29 \| #28 Limit to English \| 3031 \| | |

4. Web of Science Core Collection

| Interface: Clarivate Analytics  Editions = A&HCI , ESCI , SCI-EXPANDED , SSCI  Date of Search: 2025-10-15  Number of hits: 1132 | Field labels   - TS/Topic = title, abstract, author keywords and Keywords Plus - NEAR/x = within x words, regardless of order - * = truncation of word for alternate endings   Note: the *Exact search*-function was used for all the searches |
| --- | --- |
| \| # \| Search Query \| Results \| \| --- \| --- \| --- \| \| 1 \| TS=("family planning" OR "planned pregnan*" OR contracept* OR "fertility control" OR "unplanned pregnan*" OR "unintended pregnan*" OR "unwanted pregnan*" OR "birth spacing" OR "birth interval*" OR preconception OR "reproductive behavior") \| 121458 \| \| 2 \| TS=(childbearing OR childless* OR "Coitus Interruptus" OR "Periodic Abstinence" OR "Ovulation Inhibition" OR "Ovulation Suppression" OR Sterilization) \| 65443 \| \| 3 \| #1 OR #2 \| 180429 \| \| 4 \| TS=(econsultation* OR ecounseling OR "e health*" OR ehealth* OR "e* prescri*" OR "e therapies" OR "e therapy" OR etherap* OR "e visit*" OR evisit* OR "m health" OR mhealth OR "tele health*" OR telehealth* OR teleconsultation* OR "tele consultation*" OR "tele medicine" OR telemedicine OR telerehabilitation OR "tele rehabilitation") \| 106096 \| \| 5 \| TS=(remote* NEAR/2 (advice OR coaching OR counsel* OR consult* OR intervention* OR guid* OR rehabilitat* OR self-guid* OR support OR tailor* OR therap* OR treat* )) \| 10938 \| \| 6 \| TS=(distance NEAR/2 (advice OR coaching OR counsel* OR consult* OR intervention* OR guid* OR rehabilitat* OR self-guid* OR support OR tailor* OR therap* OR treat* )) \| 7930 \| \| 7 \| TS=(electronic* NEAR/2 (advice OR coaching OR counsel* OR consult* OR intervention* OR guid* OR rehabilitat* OR self-guid* OR support OR tailor* OR therap* OR treat* )) \| 14689 \| \| 8 \| TS=(app OR apps OR chat* OR cellphone* OR computer* OR cyber OR digital OR "e mail" OR email OR internet* OR "mobile application*" OR online OR phone* OR smartphone* OR sms OR "social media" OR technolog* OR telephone* OR "text messag*" OR video* OR virtual OR web ) \| 5860616 \| \| 9 \| TS=(twitter OR Instagram OR facebook OR tiktok OR whatsapp OR messenger) \| 448481 \| \| 10 \| #4 OR #5 OR #6 OR #7 OR #8 OR #9 \| 6297326 \| \| 11 \| TI=(“randomi$ed” OR “randomi$ed” OR “randomi$ation” OR “randomi$ation” OR placebo* OR (random* AND (allocat* OR assign*) ) OR (blind* AND (“single” OR “double” OR “treble” OR “triple”) )) OR AB=(“randomi$ed” OR “randomi$ed” OR “randomi$ation” OR “randomi$ation” OR placebo* OR (random* AND (allocat* OR assign*) ) OR (blind* AND (“single” OR “double” OR “treble” OR “triple”) )) \| 1399784 \| \| 12 \| #11 AND #10 AND #3 \| 1137 \| \| 13 \| #11 AND #10 AND #3 and English (Languages) \| 1132 \| | |
