# Supplementary material for: Tailored and Interactive Mobile Telehealth Contraceptive Counseling Compared With In-Person Care: Systematic Review and Meta-Analysis of Randomized Controlled Trials
Source: JMIR Mhealth Uhealth. 2026 Jul 16;14:e88887. doi: 10.2196/88887 (PMC13424753; doi:10.2196/88887)
Supplement: Multimedia Appendix 3 [file mhealth_v14i1e88887_app3.docx]

*
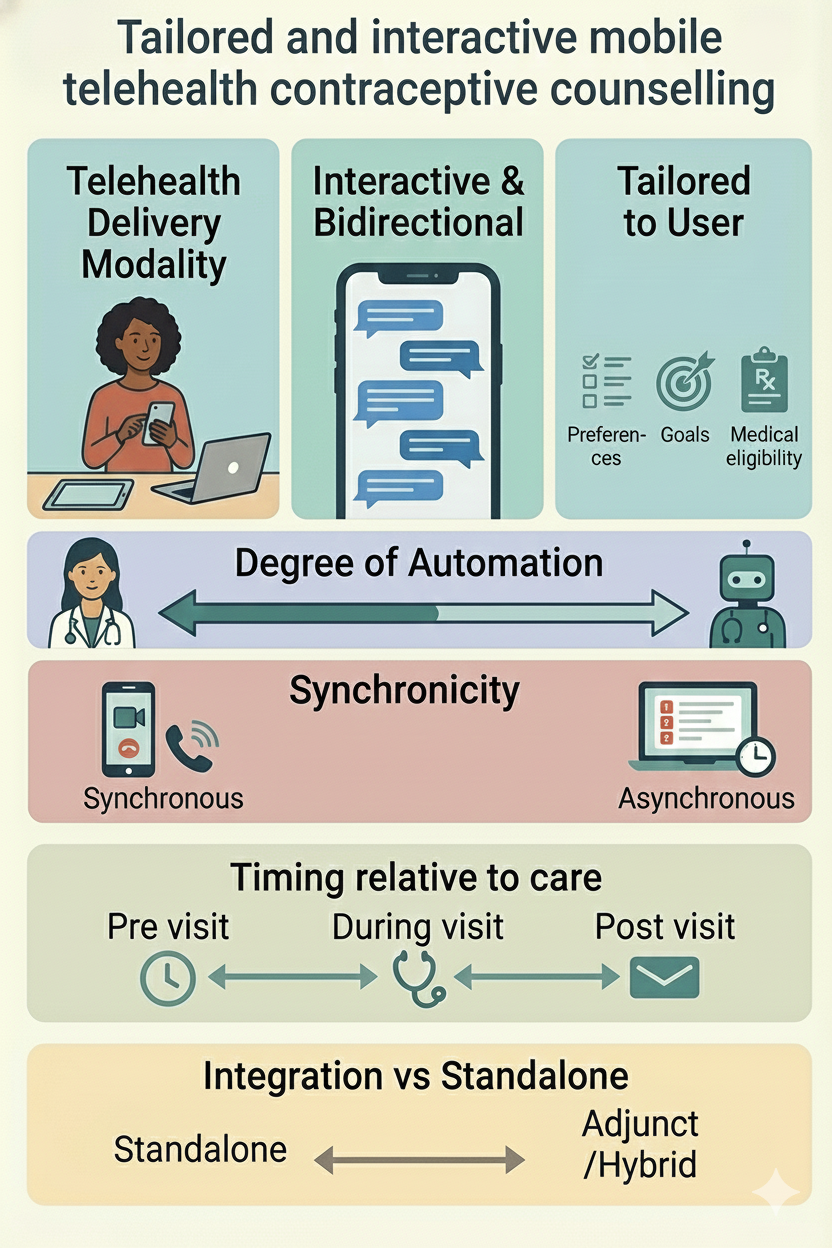
*

*Supplement Figure 1. Artificial intelligence-generated conceptual framework for our definition of TECC. Core domains: 1) Telehealth delivery modality with user interface on either phone, laptop, or tablet 2) Interactive and bidirectional communication 3) tailored to the user by preferences, reproductive goals, and/or medical eligibility. Additional service characteristics may vary by degree of automation and by real time or asynchronous delivery and response. Integration with clinical care can occur before, during, or after visits, and may be substituting (standalone) or adjunct (hybrid) to in-person counselling.*

Generator: Gemini, Google, December 8, 2025. Generator: Maja Weinryb. Prompt: Create a conceptual framework diagram titled 'Tailored and interactive mobile telehealth contraceptive counselling' featuring three vertical panels and four horizontal rows.Vertical columns: Top left: Telehealth Delivery Modality: Illustration of a holding a smartphone, with a laptop and tablet nearby. Top middle: Interactive & Bidirectional: Illustration of a message thread with chat bubbles to represent back-and-forth communication. Top right: Tailored to User: Illustrations of growing plants and icons representing user preferences, reproductive goals, and medical eligibility (checklist, target, prescription pad). Series of horizontal panels below: Panel label “Degree of Automation” illustration of a human doctor on the left and a robot doctor on the right, connected by a sliding scale to indicate varying levels of automated assistance. Panel label: “Synchronicity” icons representing 'Real-time' (chat bubbles) and 'Asynchronous' (email/envelope) communication. Panel label: Timing Relative to Clinical Care: Feature a timeline arrow spanning 'Pre-visit', 'During visit' (stethoscope icon), and 'Post-visit'.Panel label: “Relation to Clinical Care”  'Adjunct/Hybrid' on the one side and 'Substitute/Standalone' on the other.”

https://lh3.googleusercontent.com/rd-gg-dl/ABS2GSlg8r8CQqjFButQlVzdu1C8DiPhHheo_3KS3HWXBZ4sGhMLwCWRwsr3zbnawzWZvRochuqrIKXxBENHotUrXARbsezUaAvb6KXhacEw_QGdE2DMxrWt2oUitt0NhsSqIjZPrNqWjQ98Yel6UbD-t687roW3VVnyZUDiDYdIhXIIJQ0co0uXReABE8hHl6hbOHYm4Se1FWMTiBcbtX7DKAKQKbHwXTpX78PQ9HsRzVKdUmhVXWq4GmF6tf60BNikKzQz-onU2JLzoo24fsxcBLdYRX1-g_RfXF0YRogVqQjS_zGHcjFKYfXyqlywfHrNd9Zh1smxsbScscG2gcIICmGLk9JrEEn29GnJWFhJfYIv20kg2zyitm1UykpL4YpsBDbHV2zrOMBha7_Ex1HP5XivRtrLe8CB6SMJVVdagKSlRfsoV98IkCmIQy3d3XAwsIBRyf6_tsPtpcijDmfhJqp7HAHzQkJOuiMxyC2dWJBfGTSPJKyH7C88rJ8f_PAKskbZg3M6RCZAR_oTzhUVKBueFqzaQPe9C2aTQzrN0zGb_uCA_hYmz-84k9AD_CJu5Xp5jVVHrafuEErtJGDxdF7lCEFmRg9ihYqrvBcM9rRC6FLmUgAsvqoqWckXFRR1wJN_0COAWx1_wS2B0o2fTLfwSA8kuXbNqIqdDwLSg4a7Tuyk5NJ4AFwuBh5YceQnvGRY_IxtJP4qoBX77BxM2hjVP4x9XJhCe6PXvKb_raOpnA_0MdpHozO1Fy2NJ8B8uhhsP5VIgRp2hPwWpn4zsmyWBHh3qJAJwdn32qqj3WlQdmFEnQwepjXK3sOqKaBi5bpHSIjJzDta2_E52Ls8lPMFxAKNt5V0db1GJcnsk7rRk86O7_gXwa444exWee65BPTlwwrd1DcgespgHSEfhKKWquAmi4qTvq5DvdeMCBcfelWy6ZASiizlsHEgk8y4zWRWeKQe6F6CzT9YHMcOf-0pvqU9VUScx5-TOyFzFYGwwfCB3o_uzO2upLIP2C1V4sHDBz0F4gIuFLcopbyV4fwv4BIdl8btE5f0iDjWO206AUJVwpnDuzeMIjxC3DpfhNtGLiAr28yK-Tw_YGyhKzpUc14JJ5H_DlzrVSJndAZqdCzC1neVdKkETqeJWYgzFILJRghyQ2GyKHNKqbNk83Z4zTGDIcSlm1psqB5s22LkNKSW0jJCuJXVtYEwVXmxgTVHl46jDYmIPdIf0f26rxNkBGc=s1024-rj.
