# Supplementary material for: Tailored and Interactive Mobile Telehealth Contraceptive Counseling Compared With In-Person Care: Systematic Review and Meta-Analysis of Randomized Controlled Trials
Source: JMIR Mhealth Uhealth. 2026 Jul 16;14:e88887. doi: 10.2196/88887 (PMC13424753; doi:10.2196/88887)
Supplement: Multimedia Appendix 4 [file mhealth_v14i1e88887_app4.docx]

|  | **Title** | **Authors** | **Reason for exclusion** |
| --- | --- | --- | --- |
| 1 | 30. Reducing Health Disparities in Unintended Pregnancies Among Latina Adolescents Using a Patient-Centered Computer-Based Clinic Intervention | Tebb, K. and Rodriguez, F. and Pollack, L. and Puffer, M. and Adams, S. and Hwang, L. and Rico, R. and Renteria, R. and Ozer, E. and Brindis, C. and et al. | abstract with identified trial |
| 2 | A multi-media digital intervention to improve the sexual and reproductive health of female adolescent emergency department patients | Chernick, L. S. and Santelli, J. and Stockwell, M. S. and Gonzalez, A. and Ehrhardt, A. and Thompson, J. L. P. and Leu, C. S. and Bakken, S. and Westhoff, C. L. and Dayan, P. S. | wrong comparator |
| 3 | A pilot randomised controlled trial of an interactive computer-based intervention for sexual health in adolescents and young adults | Shafii, T. and Benson, S. K. and Morrison, D. M. and Hughes, J. P. and Golden, M. R. and Holmes, K. K. | wrong outcome |
| 4 | A randomized controlled trial of an intervention delivered by mobile phone app instant messaging to increase the acceptability of effective contraception among young people in Tajikistan | McCarthy, O. and Ahamed, I. and Kulaeva, F. and Tokhirov, R. and Saibov, S. and Vandewiele, M. and Standaert, S. and Leurent, B. and Edwards, P. and Palmer, M. and Free, C. | wrong comparator |
| 5 | A randomized controlled trial of an intervention delivered by mobile phone text message to increase the acceptability of effective contraception among young women in Palestine | McCarthy, O. L. and Zghayyer, H. and Stavridis, A. and Adada, S. and Ahamed, I. and Leurent, B. and Edwards, P. and Palmer, M. and Free, C. | wrong comparator |
| 6 | A randomized controlled trial to promote long-term contraceptive use among HIV-serodiscordant and concordant positive couples in Zambia | Stephenson, R. and Vwalika, B. and Greenberg, L. and Ahmed, Y. and Vwalika, C. and Chomba, E. and Kilembe, W. and Tichacek, A. and Allen, S. | wrong intervention |
| 7 | A study protocol for an mHealth, multi-centre randomized control trial to promote use of postpartum contraception amongst rural women in Punjab, Pakistan | Gul, X. and Hameed, W. and Hussain, S. and Sheikh, I. and Siddiqui, J. U. R. | protocol |
| 8 | A Trial Assessing the Effectiveness of Text Messages in Improving Continuation of Birth Control | ClinicalTrials.gov ID NCT01545609. Jenny Francis, MD,Mount Sinai Adolescent Health Center. | protocol |
| 9 | A website to help choose contraception - a pilot trial | www.isrctn.com ISRCTN13247829 Anasztazia Gubijev, Institute of Women's Health | protocol |
| 10 | Acceptability and Efficacy of a Sexual Health Texting Intervention Designed to Support Adolescent Females | Rinehart, D. J. and Leslie, S. and Durfee, M. J. and Stowell, M. and Cox-Martin, M. and Thomas-Gale, T. and Shlay, J. C. and Havranek, E. P. | wrong intervention |
| 11 | Adjunctive Social Media for More Effective Contraceptive Counseling <i>A Randomized Controlled Trial</i> | Kofinas, J. D. and Varrey, A. and Sapra, K. J. and Kanj, R. V. and Chervenak, F. A. and Asfaw, T. | wrong intervention |
| 12 | Adjunctive Social Media for More Effective Contraceptive Counseling: A Randomized Controlled Trial (vol 123, pg 763, 2014) | Kofinas, J. D. and Varrey, A. and Sapra, K. J. and Kanj, R. V. and Chervenak, F. A. and Asfaw, T. | wrong intervention |
| 13 | Adolescents' Acceptance of Long-Acting Reversible Contraception After an Educational Intervention in the Emergency Department: A Randomized Controlled Trial | Vayngortin, T. and Bachrach, L. and Patel, S. and Tebb, K. | wrong intervention |
| 14 | An interactive website to aid young women's choice of contraception: feasibility and efficacy RCT | Stephenson, J. and Bailey, J. V. and Blandford, A. and Brima, N. and Copas, A. and D'Souza, P. and Gubijev, A. and Hunter, R. and Shawe, J. and Rait, G. and Oliver, S. | duplicate |
| 15 | An Intervention Delivered by Mobile Phone Instant Messaging to Increase Acceptability and Use of Effective Contraception Among Young Women in Bolivia: Randomized Controlled Trial | McCarthy, O. L. and Aliaga, C. and Torrico Palacios, M. E. and Lopez Gallardo, J. and Huaynoca, S. and Leurent, B. and Edwards, P. and Palmer, M. and Ahamed, I. and Free, C. | wrong comparator |
| 16 | An mHealth Intervention for Pregnancy Prevention for LGB Teens: An RCT | Ybarra, M. and Goodenow, C. and Rosario, M. and Saewyc, E. and Prescott, T. | wrong intervention |
| 17 | An mHealth Trial to Promote the Use of Postpartum Contraception | ClinicalTrials.gov ID: NCT03612518  Ishaque Sheikh, MSc | protocol |
| 18 | An Unstructured Supplementary Service Data-Based mHealth App Providing On-Demand Sexual Reproductive Health Information for Adolescents in Kibra, Kenya: Randomized Controlled Trial | Macharia, P. and Perez-Navarro, A. and Sambai, B. and Inwani, I. and Kinuthia, J. and Nduati, R. and Carrion, C. | wrong intervention |
| 19 | App instant messaging to increase acceptability of effective contraception among young people in Tajikistan: results from a randomised controlled trial | McCarthy, O. and Kulaeva, F. and Tohirov, R. and Saibov, S. and Vandewiele, M. and Standaert, S. and Ahamed, I. and Leurent, B. and Edwards, P. and Palmer, M. and et al. | wrong comparator |
| 20 | Assessing Satisfaction With Contraceptive Counseling Using Telephone Versus Video Telehealth Visits | ClinicalTrials.gov: NCT05317468 Tessa Madden | wrong comparator |
| 21 | Assessing the impact of a digital job aid on clients' experience of family planning counselling and choice of long acting contraception methods | ISRCTN11040557 Laura A. Bates, Joseph P. Hicks, John Walley & Emily Robinson | wrong intervention |
| 22 | Busting contraception myths and misconceptions among youth in Kwale County, Kenya: results of a digital health randomised control trial | Gichangi, P. and Gonsalves, L. and Mwaisaka, J. and Thiongo, M. and Habib, N. and Waithaka, M. and Tamrat, T. and Agwanda, A. and Sidha, H. and Temmerman, M. and Say, L. | wrong intervention |
| 23 | Can personalized digital counseling improve consumer search for modern contraceptive methods? | Athey, Susan and Bergstrom, Katy and Hadad, Vitor and Jamison, Julian C. and Ozler, Berk and Parisotto, Luca and Sama, Julius Dohbit | wrong intervention |
| 24 | Computer-assisted provision of hormonal contraception in acute care settings | Schwarz, E. B. and Burch, E. J. and Parisi, S. M. and Tebb, K. P. and Grossman, D. and Mehrotra, A. and Gonzales, R. | wrong comparator |
| 25 | Contraceptive discontinuation and switching among couples receiving integrated HIV and family planning services in Lusaka, Zambia | Haddad, L. and Wall, K. M. and Vwalika, B. and Khu, N. H. and Brill, I. and Kilembe, W. and Stephenson, R. and Chomba, E. and Vwalika, C. and Tichacek, A. and Allen, S. | wrong study design |
| 26 | Decisions of adolescent mothers toward using etonogestrel implant: comparison between the people that knowledge from Print Media in individual counseling and counseling from video-based group counseling | <https://www.thaiclinicaltrials.org/show/TCTR20200320002>  Siraya Kitiyodom | wrong intervention |
| 27 | Design and impact evaluation of a digital reproductive health program in Rwanda using a cluster randomized design: study protocol | Nolan, C. and Packel, L. and Hope, R. and Levine, J. and Baringer, L. and Gatare, E. and Umubyeyi, A. and Sayinzoga, F. and Mugisha, M. and Turatsinze, J. and Naganza, A. and Idelson, L. and Bertozzi, S. and McCoy, S. | protocol |
| 28 | Design of a stage-matched intervention trial to increase dual method contraceptive use (Project PROTECT) | Peipert, J. and Redding, C. A. and Blume, J. and Allsworth, J. E. and Iannuccillo, K. and Lozowski, F. and Mayer, K. and Morokoff, P. J. and Rossi, J. S. | wrong comparator |
| 29 | Development and testing of an iOS waiting room "app" for contraceptive counseling in a Title X family planning clinic | Gilliam, M. L. and Martins, S. L. and Bartlett, E. and Mistretta, S. Q. and Holl, J. L. | wrong study design |
| 30 | Does the use of bedsider.org increase long-acting reversible contraception uptake in patients presenting for induced first-trimester abortion? | Sonalkar, S. and McClusky, J. and Vanjani, R. and Vragovic, O. | abstract with identified trial |
| 31 | Educational Multimedia Tool Compared With Routine Care for the Uptake of Postpartum Long-Acting Reversible Contraception in Individuals With High-Risk Pregnancies <i>A Randomized Controlled Trial</i> | Qureshey, E. J. and Chauhan, S. P. and Wagner, S. M. and Batiste, O. and Chen, H. Y. and Ashimi, S. and Ross, P. J. and Blackwell, S. C. and Sibai, B. M. | wrong intervention |
| 32 | Effect of a digital school-based intervention on adolescent family planning and reproductive health in Rwanda: a cluster-randomized trial | Hemono, R. and Gatare, E. and Kayitesi, L. and Hunter, L. A. and Packel, L. and Ippoliti, N. and Cerecero-Garcia, D. and Contreras-Loya, D. and Gadsden, P. and Bautista-Arredondo, S. and et al. | wrong intervention |
| 33 | Effect of a mobile phone intervention for female sex workers on unintended pregnancy in Kenya (WHISPER or SHOUT): a cluster-randomised controlled trial | Ampt, F. H. and Lim, M. S. C. and Agius, P. A. and L'Engle, K. and Manguro, G. and Gichuki, C. and Gichangi, P. and Chersich, M. F. and Jaoko, W. and Temmerman, M. and Stoove, M. and Hellard, M. and Luchters, S. | wrong comparator |
| 34 | Effect of cell-phone assisted postpartum counseling on the use of long-acting reversible contraceptives: a randomized controlled trial | Shaaban, O. M. and Abbas, A. M. and Saber, T. and Youness, E. and Farouk, M. | retracted,wrong intervention |
| 35 | Effect of Daily Text Messages on Oral Contraceptive Continuation <i>A Randomized Controlled Trial</i> | Castaño, P. M. and Bynum, J. Y. and Andrés, R. and Lara, M. and Westhoff, C. | wrong intervention |
| 36 | Effect of smartphone-based education on knowledge and self-care of reproductive health in married students | Vanestanagh, A. K. and Farshbaf-Khalili, A. and Esmaeilpour, K. and Jafarabadi, M. A. and Jahdi, N. S. | wrong intervention |
| 37 | Effects of postpartum mobile phone-based education on maternal and infant health in Ecuador | Unger, J. A. and Ronen, K. and Perrier, T. and DeRenzi, B. and Slyker, J. and Drake, A. L. and Mogaka, D. and Kinuthia, J. and John-Stewart, G. | wrong intervention |
| 38 | Effects of Using an Application for Postpartum Contraceptive Use in Family Planning Counseling during Pregnancy | Nurcahyani, L. and Widiyastuti, D. and Iman, A. T. and Cahyati, Y. and Fitrianingsih, Y. | wrong study design |
| 39 | Efficacy of a Digital Health Tool on Contraceptive Ideation and Use in Nigeria: Results of a Cluster-Randomized Control Trial | Babalola, S. and Loehr, C. and Oyenubi, O. and Akiode, A. and Mobley, A. | wrong intervention |
| 40 | Efficacy of a self-administered computerized counseling module in improving contraceptive method choice and continuation | Schwarz, E. B. and Burch, E. J. and Parisi, S. M. and Tebb, K. P. and Grossman, D. and Mehrotra, A. and Gonzales, R. | wrong outcome |
| 41 | Evaluating the impact of Marie Stopes International's digital family planning counselling application on the uptake of long-acting and permanent methods of contraception in Vietnam and Ethiopia: a study protocol for a multi-country cluster randomised controlled trial | Bates, L. A. and Hicks, J. P. and Walley, J. and Robinson, E. | wrong intervention |
| 42 | Evaluating the MyPath web-based reproductive decision support tool in VA primary care: Protocol for a pragmatic cluster randomized trial | Callegari, L. S. and Benson, S. K. and Mahorter, S. S. and Nelson, K. M. and Arterburn, D. E. and Hamilton, A. B. and Taylor, L. and Hunter-Merrill, R. and Gawron, L. M. and Dehlendorf, C. and Borrero, S. | protocol |
| 43 | Evaluation of a computerized contraceptive decision aid for adolescent patients | Chewning, B. and Mosena, P. and Wilson, D. and Erdman, H. and Potthoff, S. and Murphy, A. and Kuhnen, K. K. | wrong study design |
| 44 | Evaluation of a Web-based contraceptive decision aid: a randomized controlled trial | Madden, T. and Maddipati, R. and Secura, G. and Nease, R. and Politi, M. and Peipert, J. | abstract with identified trial |
| 45 | Evaluation of an mHealth SMS Dialogue Strategy to Meet Womens and Couples Postpartum Contraceptive Needs in Kenya (Mobile WACh XY): a Randomized Controlled Trial | Harrington, E. K. | protocol |
| 46 | Exploring the feasibility and effectiveness of a contraceptive counseling waiting room app | Gilliam, M. and Hebert, L. and Brown, R. and Akinola, M. and Hill, B. and Whitaker, A. and Quinn, M. | wrong intervention |
| 47 | Expression of Concern: effect of a mobile phoneassisted postpartum family planning service on the use of long-acting reversible contraception: a randomised controlled trial | The European Journal of Contraception & Reproductive Health Care, (2020), 25, (4), (264-268), 10.1080/13625187.2020.1764528) | Wrong publication type, expression of concern |
| 48 | Formal education or social electronic media-which is better in creating awareness of emergency contraception? a study on nursing students in North-East India | Bhattacharya, S. and Km, H. and Singh, L. R. and Singh, N. N. | wrong outcome |
| 49 | Improving contraceptive use among Latina adolescents: A cluster-randomized controlled trial evaluating an mHealth application, Health-E You/Salud iTu | Tebb, K. P. and Rodriguez, F. and Pollack, L. M. and Adams, S. and Rico, R. and Renteria, R. and Trieu, S. L. and Hwang, L. and Brindis, C. D. and Ozer, E. and Puffer, M. | wrong comparator |
| 50 | In the Know: A Cluster Randomized Trial of an In-person Sexual Health Education Program Integrating Digital Technologies for Adolescents | Yarger, J. and Gutmann-Gonzalez, A. and Borgen, N. and Romero, J. and Decker, M. J. | wrong outcome |
| 51 | Internet Delivered Sexually Transmitted Infection and Teen Pregnancy Prevention Program: A Randomized Trial | Kissinger, P. J. and Green, J. and Latimer, J. and Schmidt, N. and Ratnayake, A. and Madkour, A. S. and Clum, G. and Wingood, G. M. and DiClemente, R. J. and Johnson, C. | wrong comparator |
| 52 | Internet-Delivered Sexually Transmitted Infection and Teen Pregnancy Prevention Program: A Randomized Trial | Kissinger, Patricia J. and Green, Jakevia and Latimer, Jennifer and Schmidt, Norine and Ratnayake, Aneeka and Madkour, Aubrey Spriggs and Clum, Gretchen and Wingood, Gina M. and DiClemente, Ralph J. and Johnson, Carolyn | wrong comparator |
| 53 | LARC uptake in high-risk pregnancies with decision aid versus routine care: one-year postpartum follow-up | Ibarra, C. and Qureshey, E. J. and Chen, H. Y. and Wagner, S. M. and Ashimi, S. and Ross, P. J. and Blackwell, S. C. and Sibai, B. M. and Chauhan, S. P. | wrong intervention |
| 54 | Long-acting reversible contraception and satisfaction with structured contraceptive counselling among non-migrant, foreign-born migrant and second-generation migrant women: evidence from a cluster randomised controlled trial (the LOWE trial) in Sweden | Emtell Iwarsson, K. and Larsson, E. C. and Bizjak, I. and Envall, N. and Kopp Kallner, H. and Gemzell-Danielsson, K. | wrong intervention |
| 55 | Main findings from the my new options study: a randomized controlled trial of a web-based reproductive life planning intervention | Chuang, C. H. and Weisman, C. and Velott, D. and Lehman, E. B. and Moos, M. K. and Sciamanna, C. and Legro, R. and Armitage, C. and Chinchilli, V. | duplicate |
| 56 | miPlan: a Trial of miPlan Intervention vs. Standard of Care | ClinicalTrials.gov ID NCT02396602, Melissa Gilliam | protocol |
| 57 | Mobile application for information on reversible contraception: a randomized controlled trial | Sridhar, A. and Chen, A. and Forbes, E. R. and Glik, D. | wrong comparator |
| 58 | Mobile contraceptive application use in a clinical setting in addition to standard contraceptive counseling: A randomized controlled trial | Hebert, L. E. and Hill, B. J. and Quinn, M. and Holl, J. L. and Whitaker, A. K. and Gilliam, M. L. | wrong intervention |
| 59 | Mobile Link - a theory-based messaging intervention for improving sexual and reproductive health of female entertainment workers in Cambodia: study protocol of a randomized controlled trial | Brody, C. and Tuot, S. and Chhoun, P. and Swendeman, D. and Kaplan, K. C. and Yi, S. | wrong outcome |
| 60 | Mobile Phone Messaging to Improve Reproductive Health for Women Living With HIV in Kenya (Mobile WACh Empower) | ClinicalTrials.gov Identifier: NCT05285670, Alison Drake | protocol |
| 61 | Mobile phone-based postnatal follow up and maternal health outcomes for low risk mothers | Ghani, R. M. A. and Khalaf, A. | wrong outcome |
| 62 | Motivational intervention to reduce rapid subsequent births to adolescent mothers: a community-based randomized trial | Barnet, B. and Liu, J. and DeVoe, M. and Duggan, A. K. and Gold, M. A. and Pecukonis, E. | wrong outcome |
| 63 | MyNewOptions: an Online Study of Reproductive Life Planning and Contraceptive Action Planning | ClinicalTrials.gov Identifier: NCT02100124, Cynthia H Chuang | wrong comparator |
| 64 | One-Year Follow-up After a Pregnancy Prevention Intervention for LGB1 Teens: An RCT | Ybarra, M. and Rosario, M. and Saewyc, E. and Goodenow, C. and Dunsiger, S. | wrong intervention |
| 65 | Personalized contraceptive assistance and uptake of long-acting, reversible contraceptives by postpartum women: a randomized, controlled trial | Simmons, K. B. and Edelman, A. B. and Li, H. and Yanit, K. E. and Jensen, J. T. | wrong intervention |
| 66 | Plan a birth control: randomized controlled trial of a mobile health application | Sridhar, A. and Chen, A. and Glik, D. | protocol |
| 67 | Postabortion long-acting reversible contraception desire in women counselled using Bedsider.org versus standard counselling: A randomised trial | Sonalkar, S. and McClusky, J. and Vanjani, R. and Vragovic, O. and Sammel, M. D. and Borgatta, L. | wrong publication type, letter to editor |
| 68 | Promoting teen-to-teen contraceptive communication with the SpeakOut intervention, a cluster randomized trial | Tebb, K. P. and Dehlendorf, C. and Rodriguez, F. and Fix, M. and Tancredi, D. J. and Reed, R. and Brindis, C. D. and Schwarz, E. B. | wrong intervention |
| 69 | Reducing Unintended Pregnancies Through Web-Based Reproductive Life Planning and Contraceptive Action Planning among Privately Insured Women: Study Protocol for the MyNewOptions Randomized, Controlled Trial | Chuang, C. H. and Velott, D. L. and Weisman, C. S. and Sciamanna, C. N. and Legro, R. S. and Chinchilli, V. M. and Moos, M. K. and Francis, E. B. and Confer, L. N. and Lehman, E. B. and Armitage, C. J. | protocol |
| 70 | Right For Me: protocol for a cluster randomised trial of two interventions for facilitating shared decision-making about contraceptive methods | Thompson, R. and Manski, R. and Donnelly, K. Z. and Stevens, G. and Agusti, D. and Banach, M. and Boardman, M. B. and Brady, P. and Colon Bradt, C. and Foster, T. and Johnson, D. J. and Li, Z. and Norsigian, J. and Nothnagle, M. and Olson, A. L. and Shepherd, H. L. and Stern, L. F. and Tosteson, T. D. and Trevena, L. and Upadhya, K. K. and Elwyn, G. | wrong intervention |
| 71 | Short message service communication improves exclusive breastfeeding and early postpartum contraception in a low- to middle-income country setting: a randomised trial | Unger, J. A. and Ronen, K. and Perrier, T. and DeRenzi, B. and Slyker, J. and Drake, A. L. and Mogaka, D. and Kinuthia, J. and John-Stewart, G. | wrong outcome |
| 72 | Tailored health messaging improves contraceptive continuation and adherence: results from a randomized controlled trial | Garbers, S. and Meserve, A. and Kottke, M. and Hatcher, R. and Chiasson, M. A. | wrong outcome |
| 73 | Tailored intervention to increase dual-contraceptive method use: a randomized trial to reduce unintended pregnancies and sexually transmitted infections | Peipert, J. F. and Redding, C. A. and Blume, J. D. and Allsworth, J. E. and Matteson, K. A. and Lozowski, F. and Mayer, K. H. and Morokoff, P. J. and Rossi, J. S. | wrong comparator |
| 74 | The ARMADILLO text message intervention to improve the sexual and reproductive health knowledge of adolescents in Peru: Results of a randomized controlled trial | Perez-Lu, J. E. and Guerrero, F. and Carcamo, C. P. and Alburqueque, M. and Chiappe, M. and Hindin, M. J. and Habib, N. and Say, L. and Gonsalves, L. and Bayer, A. M. | wrong outcome |
| 75 | The effect of the bedsider.org web site on contraceptive use within an urban gynecology clinic | Jamshidi, R. M. and Robinson, J. and Burke, A. E. | abstract with identified trial |
| 76 | The effectiveness of a digital shared decision-making tool in hormonal contraception during clinical assessment: study protocol of a randomized controlled trial in Spain | de Molina-Fernandez, M. I. and Raigal-Aran, L. and de la Flor-Lopez, M. and Prata, P. and Font-Jimenez, I. and Valls-Fonayet, F. and March-Jardi, G. and Escuriet-Peiro, R. and Rubio-Rico, L. | wrong population |
| 77 | The Effects of Text Messaging for Increasing the Rate of long-acting Reversible Contraception use in Teenage Pregnancy: a Randomized Controlled Trial | Meesin, K. and Intarat, N. and Temeiam, N. and Kingsawad, K. | wrong intervention |
| 78 | The impact of a direct to beneficiary mobile communication program on reproductive and child health outcomes: a randomised controlled trial in India | LeFevre, A. E. and Shah, N. and Scott, K. and Chamberlain, S. and Ummer, O. and Bashingwa, J. J. H. and Chakraborty, A. and Godfrey, A. and Dutt, P. and Ved, R. and Mohan, D. and Kilkari Impact Evaluation, T. | wrong intervention |
| 79 | The Impact of Get It? on Long-Acting Reversible Contraception Use Among Adolescents and Young Adults | Patel, Pooja R. and Nandigam, Likhita and Thompson, Jada and Abacan, Allyssa and Raphael, Meghna | wrong intervention |
| 80 | Use Of Youth-Centered Mobile Health Application, Health-E You/Salud iTu, To Reduce Disparities In Contraceptive Knowledge, Access And Unintended Pregnancy Among Sexually Active Latina Adolescents | Tebb, K. and Trieu, S. and Rodriguez, F. J. and Pollack, L. and Adams, S. and Ricco, R. and Renteria, R. and Hwang, L. and Ozer, E. and Sandoval, D. and et al. | abstract with identified trial |
| 81 | Using mHealth to Promote Post-Menstrual Regulation Contraceptive Uptake and Continuation in Bangladesh | ClinicalTrials.gov Identifier: NCT02579785, Kathryn Anderson | protocol |
| 82 | UTAH: Using Telemedicine to improve early medical Abortion at Home: a protocol for a randomised controlled trial comparing face-to-face with telephone consultations for women seeking early medical abortion | Reynolds-Wright, J. J. and Norrie, J. and Cameron, S. T. | protocol |
| 83 | Video Versus Conversational Contraceptive Counseling During Maternity Hospitalization | ClinicalTrials.gov Identifier: NCT03400449, Maureen Baldwin | wrong intervention |
| 84 | Acceptability of digital health intervention during pregnancy to inform women about postpartum contraception (DIGICAP): a pilot randomised controlled study. | Cooper, Michelle and Free, Caroline J and Kuan, Kevin Ka-Wing and McCabe, Karen and Osei-Asemani, Emmanuela and Opondo, Charles and Cameron, Sharon | wrong study design |
| 85 | Effectiveness of an Interactive Mobile Health Intervention (IMHI) to enhance the adoption of modern contraceptive methods during the early postpartum period among women in Northeast Ethiopia: A cluster Randomized Controlled Trial (RCT). | Cherie, Niguss and Wordofa, Muluemebet Abera and Debelew, Gurmesa Tura | wrong intervention |
| 86 | Interactive Mobile Phone Application for Better Family Planning Services in Jordan: a Quasi-experimental Study | Tailakh, SN and Basha, ASH and Kasassbeh, RS | wrong outcome |
