# Supplementary material for: Tailored and Interactive Mobile Telehealth Contraceptive Counseling Compared With In-Person Care: Systematic Review and Meta-Analysis of Randomized Controlled Trials
Source: JMIR Mhealth Uhealth. 2026 Jul 16;14:e88887. doi: 10.2196/88887 (PMC13424753; doi:10.2196/88887)
Supplement: Multimedia Appendix 5 [file mhealth_v14i1e88887_app5.docx]

| **Author and year** | **Study arms** | **Follow-up schedule** | **Primary outcome(s)** | **Secondary outcome/s** | **Main findings/results** | **Authors’ conclusions** |
| --- | --- | --- | --- | --- | --- | --- |
|  |  |  |  |  |  |  |
| **Reiss 2019** (Bangladesh, public post-menstrual regulation services) | Interactive voice messages vs standard care | 2 weeks and 4 months | LARC use at 4 months | LARC use at 2w, ECM use at 2w and 4 months, repeat pregnancy and MR, contraceptive discontinuation, adverse effects including intimate partner violence (IPV) | No effect on contraceptive use; increased reports of IPV | Telehealth contraception messages may carry risk of unintended harm (IPV); caution required |
|  |  |  |  |  |  |  |
| **Stephenson 2020** (UK, sexual and reproductive health clinics and online booking system) | Website with interactive decisions aid vs standard care | 3 and 6 months | LARC use and satisfaction with method at 6 months | Contraceptive use at 6 months;  change in method from baseline to 6 months; pregnancy  by 6 months, sexually transmitted  infection at 3 or 6 months, adverse effect (any good or bad effect from being in the trial free text question) | No difference in LARC use or satisfaction; no difference in adverse effects, STI or pregnancy; positive user feedback | Digital tools support choice but structural/access barriers must be addressed |
|  |  |  |  |  |  |  |
| **Harrington 2019** (Kenya, public hospitals, antenatal to postpartum) | SMS messages from 28 w of gestation to 6 months postpartum  Vs Standard antenatal and postpartum care | 6 weeks, 14 weeks, 6 months postpartum | ECM use at 6 months postpartum | ECM use at 6w and 14 weeks, any contraceptive use, exclusive breastfeeding, satisfaction with method, method discontinuation at 6 months, time to method initiation, dual contraceptive use, | ECM use significantly higher in intervention arm at 6 months (aRR 1.26, p=0.02) but not earlier time points | Intervention can increase postpartum ECM use. May have larger effect on continuation than initiation. Optimal timing needs investigation. |
|  |  |  |  |  |  |  |
| **Garbers 2012** (USA, urban family planning clinics) | Tablet based decision aid vs standard care | End-of-visit | Effectiveness of method chosen at end of visit | Satisfaction with module | Increased choice of ECM in both intervention arms ( tailored 75%, generic 78%) vs control (65%) | Computer-based decision aids can improve contraceptive method selection in low-literacy groups |
| **Aksut 2024** (Turkey, antenatal care during COVID-19) | Video counselling vs standard care | Intervention: immediately after the second video call  Controls: Two weeks after baseline | Knowledge about family planning and satisfaction with family planning services |  | Significant increases in family planning knowledge and satisfaction | Telemedicine counselling improved knowledge and satisfaction; promising beyond pandemic use |
|  |  |  |  |  |  |  |
| **Smith 2015** (Cambodia, Marie Stopes clinics post-abortion) | Interactive voice messages vs  Standard care | 4 and 12 months | Use of ECM at 4 and 12 months | LARC use, repeat pregnancy, repeat abortion, method continuation, road traffic accidents, domestic abuse related to the intervention | Increased use of ECM at 4 months, not at 12 months; increased LARC use | Intervention increased short-term ECM use and increased LARC use throughout the study period |
|  |  |  |  |  |  |  |
|  |  |  |  |  |  |  |
| **Dehlendorf 2019** (USA, urban family planning, public health, college student health center and hospital outpatient clinic) | Tablet optimized-website  Vs standard care | 4 and 7 months | 7-month continuation of chosen method | Continuous use of any method, knowledge, decision quality, method choice, satisfaction with counselling | No difference between groups on choice or use of EMC or continuation; intervention improved knowledge, decision quality and patient experience | Intervention improved quality of care and informed decision making and knowledge but not method use or continuation |
|  |  |  |  |  |  |  |
|  |  |  |  |  |  |  |
|  |  |  |  |  |  |  |
| **Madden**  (USA, Obstetrics/Gynaelcologi clinics at academic center) | Tablet-based decision aid vs standard care | Immediately-post intervention | Change in decisional conflict before and after visit | Choice of contraceptive method, preferences, satisfaction with visit, decisional conflict | No difference between intervention group and control on: decisional conflict, choice of contraceptive method, satisfaction | The decision aid showed no effect but may be more useful in settings with limited contraceptive counselling. |
|  |  |  |  |  |  |  |
| **Reynolds-Wright**  (UK, Public abortion care centre Edinburgh) | Telephone counselling vs standard care | 2 weeks | Efficacy of medical abortion, defined as  complete abortion without surgical intervention | Satisfaction with consultation, preparedness, unscheduled contact with care, complication rate, time spent in clinical contact, choice of ECM and LARC | No differences for all outcomes except shorter time spent in clinic for intervention group. | Intervention appears effective safe and acceptable with less time spent in clinic, but study underpowered due to early cessation and could thus not establish non-inferiority |
|  |  |  |  |  |  |  |
