# Supplementary material for: Tailored and Interactive Mobile Telehealth Contraceptive Counseling Compared With In-Person Care: Systematic Review and Meta-Analysis of Randomized Controlled Trials
Source: JMIR Mhealth Uhealth. 2026 Jul 16;14:e88887. doi: 10.2196/88887 (PMC13424753; doi:10.2196/88887)

| **Study (Setting)** | **Mode of delivery / user interface** | **General intervention description** | **INTERVENTION CORE COMPONENTS** | | **INTERVENTION MODIFIERS** | | | **Comparator** | **TECC relation to clinical care** | **Nature of User Interaction with TECC** |
| --- | --- | --- | --- | --- | --- | --- | --- | --- | --- | --- |
|  |  |  | **Tailoring** | **Interactivity** | **Degree of automation / Provider Involvement** | **Timing relative to clinical care** | **Duration** |  |  |  |
| **Reiss 2019** (Bangladesh, public post-menstrual regulation services) | Mobile phone.  Interactive voice  messages | Pre-recorded interactive voice messages delivered method information and addressed concerns; designed to support post-procedure contraceptive use | Messages tailored to contraceptive method chosen at MR procedure (and changed if switch of method)  Optional individual counselling call | User navigated voice menu. Could request connection to call center for further counselling | Automated messages; Nurse available through call center upon user request | Delivered after in-person menstrual regulation procedure and CC | 4 months, 7 weekly messages and then 4 fortnightly messages | Standard post-menstrual regulation contraceptive counselling without digital follow-up | Hybrid/Complementary  All participants received standard care | Automated system delivers information; optional delayed synchronous contact with provider |
| **Stephenson 2020** (UK, sexual and reproductive health clinics and online booking system) | Mobile-optimized website  Interactive decision aid and educational content | Mobile-optimized website with videos, FAQs, and a tailored decision aid resulting in three matched methods based on user preferences regarding seven contraception attributes | Tailored method suggestions based on user input | User interacts with dynamic decision aid and receives real-time tailored feedback from system | Automated; self-directed use with no provider interaction | Used before clinical appointment; designed to inform user choice ahead of in-person visit | One-time use | Usual care; website access offered after study follow-up | Hybrid/Complementary  (Independent use informing in-person consultation. all participants received standard care) | Fully automated real-time interaction; no human input |
| **Harrington 2019** (Kenya, public hospitals, antenatal to postpartum) | Mobile phone  SMS messages | SMS messages included contraceptive content and prompted user responses. Messages were tailored and multilingual. Nurses engaged in real-time or asynchronous dialogue. Optional enrolment of partner | Messages tailored to gestational age /postpartum stage, contraceptive method choice, language, and user responses | Users could initiate and respond to messages; nurse responded accordingly | Automated messaging and prompted provider response | Initiated at enrolment 28 weeks of pregnancy; continued 6 months postpartum to support contraceptive use. Synchronous/ Asynchronous | Weekly automated messages from 28 weeks gestation to 6 months postpartum | Standard antenatal and postpartum care with no SMS follow-up | Hybrid/Complementary  All participants received standard care | Bidirectional interaction with provider via SMS, asynchronous responses |
| **Garbers 2012** (USA, urban family planning clinics) | Touchscreen laptop | Audio computer assisted self-interview (ACASI) module about contraception and medical conditions. Resulted in tailored recommendation based on user input green/yellow/red | Resulting in printed tailored method recommendations Green = aligning with life goals and best pregnancy prevention  Yellow = less effective methods or less good fit  Red = medically contraindicated methods | User navigated ACASI system. Using headphones and touchscreen, no reading or typing needed. | Automated system  User encouraged to present tailored printout for use during in-person counselling session | Used by patient in waiting room before in-person counselling session | One-time use | Control group completed demographic questions on laptop and received printed materials on available contraception | Hybrid / Complementary  All participants received standard care | Real-time automated interaction with personalized output |
| **Aksut 2024** (Turkey, antenatal care during COVID-19) | Mobile phone  What’sApp video call | Video calls with counsellors using handbook for counselling and showing contraceptive methods | Information tailored in real-time call | Live, synchronous counselling session. User prompted to ask questions | No automation, fully human delivered synchronous counselling via video call | Provided in third trimester of pregnancy, two weeks apart | Approximately 50 minutes; twice | Standard family planning counselling during in-person antenatal visit at 24 weeks of gestation | Hybrid/Complementary  All participants received standard care | Synchronous interaction with provider; fully human-delivered via digital channel |
| **Smith 2015** (Cambodia, Marie Stopes clinics post-abortion) | Mobile phone  Interactive voice messages | Interactive voice messages with optional additional phone counselling after clinical abortion service, designed to support post-abortion contraceptive use | Messages customized to method choice; optional method use reminders, optional call back from counsellor | User navigated messages and could trigger follow-up support from nurse | Automated content; counsellor callback upon request | Delivered after abortion care including CC | Started within one week from abortion up to 3 months after. Fortnightly messages | Routine post-abortion counselling; follow-up contact information | Hybrid/Complementary  All participants received standard care | Automated content with option for human-delivered follow-up |
| **Dehlendorf 2019** (USA, urban family planning clinics) | Tablet-optimised website  Tablet | Decision aid with interactive educational method information and survey eliciting user preferences, reproductive life goals and certain medical eligibility criteria. Results in a summary sheet with recommendations based on user input, intended to use for shared decision-making during in-person consultation. | Tailored method generated from user input | Interactive: user responds to questions/statements, option for free text questions. | Automated tool with real-time system feedback;  No provider interaction during tool use. Resulting summary printed for use in in-person consultation | Completed in waiting room immediately before clinic visit | One-time use | Standard counselling | Hybrid/complementary  All participants received standard care | Real-time system feedback; no direct provider interaction during tool use |
| **Madden** | Tablet-based tool  Tablet | Tablet-based decision aid designed to elicit user preferences and result in top three tailored recommendations based on user input. Includes medical and reproductive history, prior contraceptive use, reproductive plans and contraceptive preferences. | Tablet and printed handout listing the user's top three preferences, three suggested methods, other appropriate methods, and any medically contraindicated methods. | Interactive: user ranking contraceptive preferences | Automated system with | Prior to in-person clinic visit | One-time | Demographic and reproductive survey on tablet; received a non-tailored handout on reproductive health care (e.g., cervical cancer and STI screening) | Hybrid/complementary  All participants received standard care | Real-time automated interaction with personalised printed output; no direct provider interaction during tool use. |
| **Reynolds-Wright** | Phone  Phone call | Phone call for abortion consultation and discussion of postabortion contraception | Information tailored in real-time call | Live, synchronous counselling session | No automation, fully human delivered synchronous counselling | Instead of in-person counselling. Followed by in-person ultrasound and medication provision | One-time | Standard in-person counselling | Standalone/replacing parts of in-person care |  |


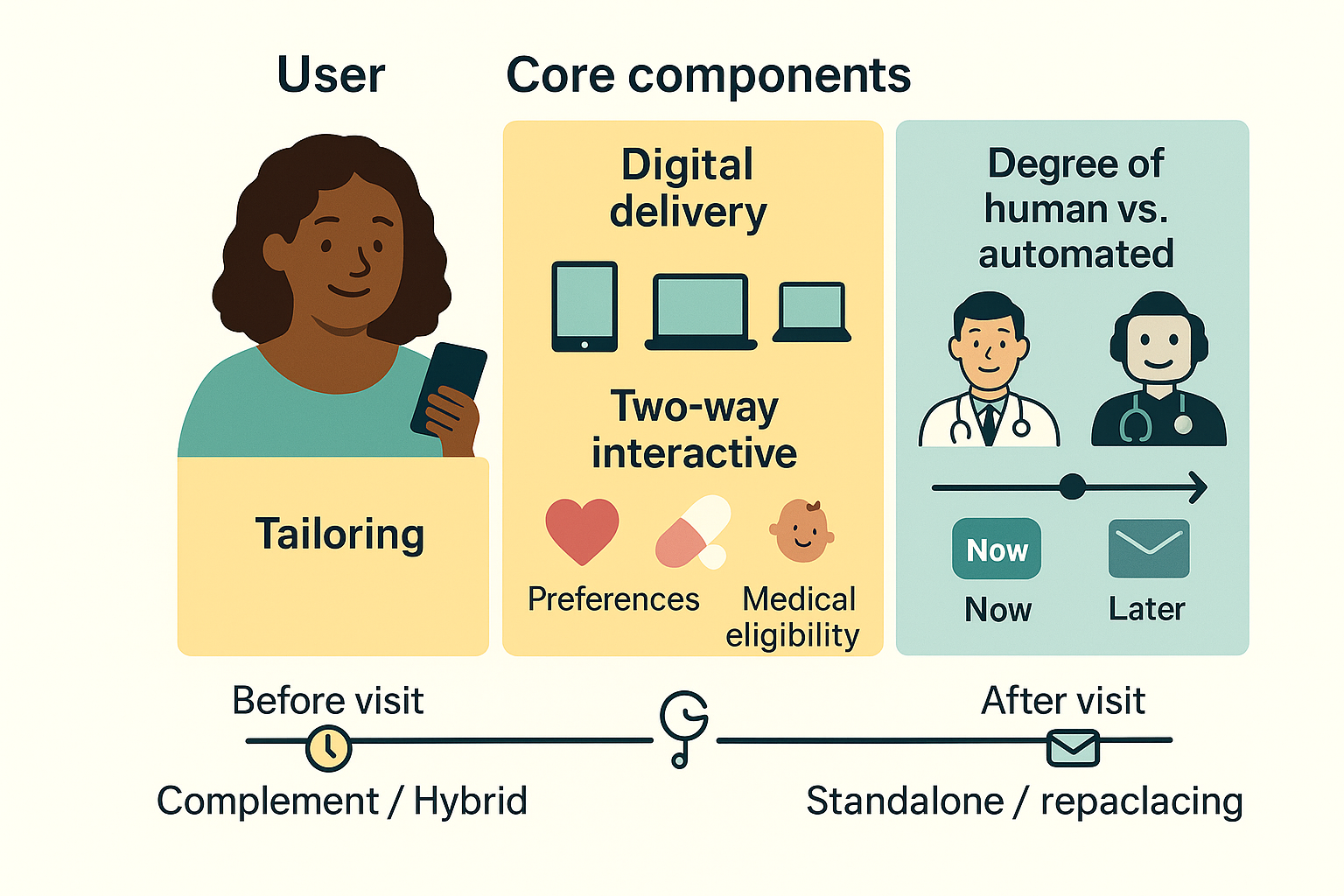

Supplement: Multimedia Appendix 6 [file mhealth_v14i1e88887_app6.docx]
