# Supplementary material for: Tailored and Interactive Mobile Telehealth Contraceptive Counseling Compared With In-Person Care: Systematic Review and Meta-Analysis of Randomized Controlled Trials
Source: JMIR Mhealth Uhealth. 2026 Jul 16;14:e88887. doi: 10.2196/88887 (PMC13424753; doi:10.2196/88887)
Supplement: Multimedia Appendix 7 [file mhealth_v14i1e88887_app7.docx]

| **TECC compared to standard care for CC** | | | | | | | |
| --- | --- | --- | --- | --- | --- | --- | --- |
| **Certainty assessment** | | | | | | |  |
| **Participants (studies) Follow-up** | **Risk of bias** | **Inconsistency** | **Indirectness** | **Imprecision** | **Publication bias** | **Overall certainty of evidence** |  |
|  |  |  |  |  |  |  |  |
| **Use of ECM<6 months** | | | | | | | |
| 2180 (4 RCTs)^a^ | not serious | serious^b^ | not serious | serious^c^ | none | ⨁⨁◯◯ Low^b,c^ |  |
| **Use of ECM at 6-12 months** | | | | | | | |
| 2042 (4 RCTs)^a^ | serious^d^ | not serious | not serious | serious^e^ | none | ⨁⨁◯◯ Low^d,e^ |  |
| **Use of LARC <6 months** | | | | | | | |
| 1203 (2 RCTs) | not serious | serious^f^ | not serious | very serious^g^ | none | ⨁◯◯◯ Very low^f,g^ |  |
| **Use of LARC 6-12 months** | | | | | | | |
| 1042 (2 RCTs)^a^ | serious^h^ | serious^i^ | not serious | very serious^j^ | none | ⨁◯◯◯ Very low^h,i,j^ |  |
| **Choice of ECM** | | | | | | | |
| 2590 (4 RCTs) | serious^k^ | serious^l^ | not serious | serious^c^ | none | ⨁◯◯◯ Very low^c,k,l^ |  |
| **Choice of LARC** | | | | | | | |
| 336 (2 RCTs) | serious^h^ | not serious | not serious | very serious^m^ | none | ⨁◯◯◯ Very low^h,m^ |  |
| **Satisfaction with counselling <6 months** | | | | | | | |
| 749 (2 RCTs) | very serious^n^ | serious^o^ | not serious | serious^p^ | none | ⨁◯◯◯ Very low^n,o,p^ |  |
| **Use of ECM <6 months in LMIC or Low RoB** | | | | | | | |
| 1431 (3 RCTs)^a^ | not serious | serious^q^ | not serious | very serious^r^ | none | ⨁◯◯◯ Very low^q,r^ |  |

**CI:** confidence interval; **RR:** risk ratio

#### Explanations

a. Numbers calculated from percentages, rounded to whole

b. Heterogeneity I-squared = 0,76

c. OIS adequate. Downgraded for imprecision because effect estimate CI overlaps no effect

d. Moderate RoB in 2/4 studies

e. OIS adequate but lower bound of effect estimate confidence interval 1,002, fails to reliably exclude no effect

f. Heterogeneity I-squared = 0.95

g. OIS not met. Very wide CI of effect estimate. Lower bound below one, fails to exclude no effect.

h. Moderate RoB in 1/2 studies

i. Heterogeneity I-squared = 0,86

j. Results not pooled. Smith very wide CI of effect estimate, Stephenson lower bound of CI fails to exclude no effect.

k. High and moderate RoB in 2/2 studies respectively

l. Heterogeneity I-squared = 0,53

m. Total sample sizes small. Lower bound of effect estimates CI fails to exclude no effect.

n. High RoB in 2/2 studies

o. Data cannot be pooled due to different measurement methods. One study showed no difference and the other study suggested higher satisfaction in intervention group.

p. Data cannot be pooled. Lower bound of effect estimate CI (Dehlendorf) fails to exclude no effect.

q. Heterogeneity I-squared = 0.7

r. Wide confidence interval of effect estimate. Lower bound below one.
